# Adverse Effects of Surgically Accelerated Orthodontic Techniques: A Systematic Review

**DOI:** 10.3390/children9121835

**Published:** 2022-11-27

**Authors:** Ioanna Pouliezou, Angeliki Xenou, Konstantina Vavetsi, Anastasia Mitsea, Iosif Sifakakis

**Affiliations:** 1 Department of Orthodontics, School of Dentistry, National and Kapodistrian University of Athens, 11527 Athens, Greece; 2 Department of Periodontics, School of Dentistry, National and Kapodistrian University of Athens, 11527 Athens, Greece; 3 Department of Oral Diagnosis & Radiology, School of Dentistry, National and Kapodistrian University of Athens, 11527 Athens, Greece

**Keywords:** accelerated tooth movement, orthodontics, corticotomy, corticision, piezocision, micro-osteoperforetions, lasercision, adverse effects, systematic review

## Abstract

Evidence on the potential adverse effects of surgically accelerated orthodontic techniques is scarce. The aim of this review was to evaluate the available scientific evidence regarding the adverse effects on periodontium, tooth vitality, and root resorption, associated with these surgical procedures in children, adolescents, and adults. The reporting of this review was based on the PRISMA2020 guidelines. Seven databases and three registers were searched for randomized clinical trials (RCTs) and controlled clinical trials (CCTs) published up to 22 June 2022. Hand searching of the reference lists of the included studies was also performed. The quality of the evidence was assessed with the Cochrane risk of bias and ROBINS-I tools. A total of 887 records were initially screened. Finally, 33 RCTs (713 patients), six CCTs (103 patients), and six ongoing protocols were eligible for this systematic review. The current review indicated that there are no significant adverse effects of surgically accelerated orthodontic techniques on periodontium, root length, or tooth vitality. High-quality clinical trials with less risk of bias should be conducted to allow reliable conclusions regarding the adverse effects of the surgical procedures associated with the acceleration of orthodontic treatment on children, adolescents, and adults.

## 1. Introduction

### 1.1. Rationale

Conventional orthodontic treatment on average requires less than 2 years to complete when fixed appliances are used to treat moderate to severe malocclusion [1]. Prolonged orthodontic treatment may result in several adverse effects, such as pain and discomfort, dental caries, gingival recession, and apical root resorption [2,3]. Shorter treatment time would benefit both children and adult patients, limiting discomfort, and would reduce the prevalence of iatrogenic adverse side effects [4,5,6,7]. Hence, orthodontists and patients alike are interested in techniques that can accelerate tooth movement and reduce treatment time [8].

Several methods to accelerate orthodontic tooth movement (OTM) have been proposed in order to shorten the orthodontic treatment, including specific bracket types [9], pharmacological approaches, such as the use of prostaglandins, interleukins, leukotrienes, vitamin D and platelet-rich plasma [10], photobiomodulation [11], and low-intensity laser irradiation [12]. According to research, surgical procedures have the best potential reduction in treatment time yet are limitedly applied due to their aggressiveness [13].

In 1959, corticotomy-assisted orthodontic treatment (CAOT) was introduced as an intervention to accelerate tooth movement [14]. The acceleratory impact of corticotomy was associated with a demineralization/remineralization process called the regional accelerated phenomenon (RAP) [15]. Frost initially defined RAP as a local bone mineral density reduction and bone remodeling process [16].

Corticotomy techniques are relatively invasive as full-thickness mucoperiosteal flaps are required, and, therefore, a certain morbidity for the patient is expected, including pain, swelling [17], and the minimum loss of alveolar bone as well as attached gingiva [18]. This could be an explanation for their restricted application from orthodontists in routine clinical practice. Consequently, in the past few years, minimally invasive corticotomy procedures have been suggested, such as corticision [19], piezocision [20], micro-osteoperforations (MOPs) [21], and laser-assisted flapless corticotomy (lasercision) [22].

Although the majority of the published evidence did not report any adverse effects, some papers point out a small degree of periodontal injury [15,17,23]. There have been few attempts to thoroughly examine the existing literature regarding the adverse effects of surgically assisted accelerated orthodontic techniques. Current evidence-based records have reviewed only some of these techniques, mainly corticotomy [7,24,25,26,27,28]; however, a systematic analysis of all alternative techniques and their adverse effects is still not available.

### 1.2. Objectives

The objective of the current systematic review is to critically evaluate and comprehensively summarize the available evidence concerning the potential detrimental effects of all types of surgically accelerated orthodontic techniques on periodontium, root length/resorption, and tooth vitality.

## 2. Materials and Methods

### 2.1. Design

The present systematic review was performed according to the Cochrane Handbook for Systematic Reviews of Interventions version 6.2.0 [29], and the PRISMA statement (Preferred Reporting Items for Systematic Reviews and Meta-Analysis) was followed for reporting [30,31]. The protocol was registered with the PROSPERO (international prospective register of systematic reviews) under the registration number: CRD42022264574 (https://www.crd.york.ac.uk/prospero/display_record.php?ID=CRD42022264574, accessed on 23 October 2022). The review followed the registered protocol without deviation from its original design.

### 2.2. Eligibility Criteria

Inclusion and exclusion criteria were implemented in accordance with the Participants, Interventions, Comparisons, Outcomes, and Study design (PICOS) framework.

#### 2.2.1. Types of Participants

Healthy patients with any type of malocclusion and no history of previous orthodontic or periodontal treatment. No age, gender, or ethnic group limitations were applied.

#### 2.2.2. Types of Interventions

Any sort of orthodontic treatment with fixed appliances (extraction and non-extraction cases) facilitated by surgically accelerated orthodontic techniques to accelerate tooth movement (i.e., corticotomy, corticision, piezocision, micro-osteoperforations, lasercision/laser-assisted flapless corticotomy).

#### 2.2.3. Comparisons

Patients undergoing orthodontic treatment with fixed appliances with no further intervention to accelerate tooth movement. A second comparison was made using alveolar corticotomy as the control group and minimally invasive surgical interventions (corticision, piezocision, micro-osteoperforations, and lasercision) as the test group.

#### 2.2.4. Types of Outcome Measures

Adverse side effects on periodontium (probing depth, gingival index, plaque index, gingival bleeding, gingival recession, attachment loss, bone resorption, tooth mobility, dehiscence, furcation defect, and fenestration formation) and tooth vitality, as well as potential root resorption.

#### 2.2.5. Study Design

Only RCTs and CCTs in humans that investigated the relationship between surgically assisted accelerated orthodontic techniques (i.e., alveolar corticotomy, corticision, piezocision, micro-osteoperforations, and lasercision) and detrimental effects on periodontal tissues, root length, and tooth vitality were included. Studies based on the split-mouth, two-arm, and multi-arm designs were also eligible. For multi-arm studies, the experimental groups that met the criteria of the systematic review were included. The follow-up time was not taken into account.

#### 2.2.6. Exclusion Criteria

The following publications were excluded: in vitro, animal, histological, retrospective studies, case reports/series, systematic/literature reviews, technique description articles, abstracts only, opinion pieces, studies with ineligible outcomes with this review or uncompleted orthodontic treatments, and studies including other surgical procedures that were not primarily intended to accelerate orthodontic treatment, accelerated orthodontics interventions involving LeFort osteotomies, or orthognathic surgery.

### 2.3. Information Sources

The following electronic databases were comprehensively searched up to 2 October 2021: PubMed, Medline (via EBSCOhost), Scopus, Web of Science, Cochrane Library, ScienceDirect, and Google Scholar. ClinicalTrials.gov, Open Grey, and ISRCTN were screened up to 30 October 2021. The search was updated on 22 June 2022.

### 2.4. Search Strategy

The systematic search was conducted by two examiners (I.P., A.X.) using appropriate medical subject headings (MeSH) and free text words. The search was restricted to articles written in the English language and published from January 2006–June 2022. Details of the complete electronic search strategy are provided in Supplementary Table S1. ScienceDirect was used as an adjunctive search database to identify additional eligible studies with the first 100 relevant results to be considered for inclusion. A partial grey literature search was conducted by using Google Scholar and was limited to the first 100 most relevant articles. Studies from grey literature, defined as theses, dissertations, and unpublished studies were retrieved through ClinicalTrials.gov, Open Grey, and the ISRCTN registry. The reference lists of selected articles and relevant reviews were screened for any possible related studies which may have not been discovered by the electronic search.

### 2.5. Study Selection

The studies retrieved from all databases and registers were cross-checked for duplicates. The study selection was accomplished in two phases. In the first phase, two reviewers (I.P., A.X.) independently screened the titles and abstracts which were identified by all electronic databases regarding accelerated orthodontic techniques and their side effects. In case of disagreement on which articles to screen with the full text, a consensus was reached by discussion. If necessary, the final decision was made after consultation with a third reviewer (A.M.). In the second phase, full-text articles were assessed independently by two reviewers (I.P., A.X.) for inclusion. Any disagreement concerning full text inclusion was resolved with discussion and, if necessary, with the consultation of the third reviewer (A.M.) until consensus was reached.

### 2.6. Data Collection and Data Items

The same two review authors (I.P., A.X.) collected the data independently in a customized and pre-defined data extraction table. Extracted data were compared, and in case of discrepancies, a consensus was reached through discussion and the re-examination of the studies. The data extraction form included the following items: general information (authors’ names, publication year, and study setting); methods (study design, treatment comparison groups, and outcome assessment method); participants (sample size, gender, age, and malocclusion characteristics); intervention (type, site, and technical aspects of intervention); accelerated orthodontic aspects (details of surgical techniques, alveolar bone augmentation procedures and materials, type of movement, appliance characteristics and biomechanics and orthodontic adjustments’ frequency, and follow-up time) and outcomes (outcomes stated, outcome measurements’ methods).

### 2.7. Risk of Bias Assessment in Included Studies

The quality assessment of the included studies was carried out by two reviewers (I.P., A.X.) independently, using the revised Cochrane Risk of Bias (RoB 2.0) tool for randomized trials [32] and Risk Of Bias In Non-randomized Studies-of Interventions (ROBINS-I) tool for non-randomized trials [33]. In case of disagreement, the two authors thoroughly discussed until a consensus was reached. The overall judgment of the risk of bias (low, some concerns, high in RoB 2.0; low, moderate, serious, critical, no information in ROBINS-I) for each study was dictated by the highest RoB level in any of the domains that were assessed.

### 2.8. Effect Measures and Data Synthesis

The primary outcome of this systematic review was to investigate the effect of the surgical techniques used to accelerate orthodontic tooth movement on periodontal tissues, root length, and tooth vitality. Variables extracted from each article were the following: author and year of publication, study design, sample size, intervention type, treatment comparison, outcome assessment method, outcome of results, and follow-up time.

A detailed narrative description of the findings (qualitative data analysis) was pre-planned to be employed if significant clinical and methodological heterogeneity of the included studies was detected.

## 3. Results

### 3.1. Study Selection

**Figure 1:**
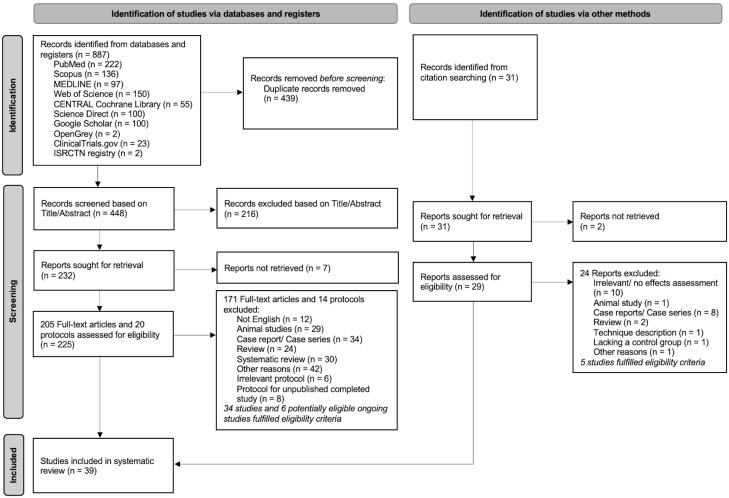
PRISMA 2020 flow diagram for new systematic reviews which included searches of databases, registers, and other sources.

In total, 887 records were retrieved from the seven databases and three registers. A total of 31 records were identified through citation searching. Duplicate references were removed, and a total of 448 articles were thoroughly screened. The titles and abstracts were assessed for eligibility, followed by the elimination of all papers not fulfilling the inclusion criteria. Seven records could not be retrieved for full-text evaluation and were removed. Therefore, 225 potentially related trials (205 articles and 20 registry entries for ongoing studies) remained for further examination. After reviewing the full-text articles and in line with the inclusion and exclusion criteria, 171 completed studies and 14 ongoing studies were excluded. More information about the studies that potentially could meet the inclusion criteria, but were excluded, can be found in Supplementary Table S2. Finally, 39 articles and 6 potentially eligible ongoing studies were included for qualitative analysis. A PRISMA2020 flow diagram is shown in Figure 1 [30].

### 3.2. Study Characteristics

Out of the 39 included studies, 33 were RCTs [34,35,36,37,38,39,40,41,42,43,44,45,46,47,48,49,50,51,52,53,54,55,56,57,58,59,60,61,62,63,64,65,66], from which 17 had a split-mouth study design [34,37,41,45,48,53,55,56,57,58,59,60,62,64,65,66] and 6 were CCTs [22,67,68,69,70,71], from which 5 had a split-mouth study design [22,67,69,70,71]. Among these 39 trials, five articles evaluated corticotomy-facilitated orthodontics [34,35,37,67,71], two tested PAOO [38,39], two studied the effects of corticision [43,44], eleven investigated piezocision [40,48,49,50,51,52,53,54,55,68,70], eight tested accelerated tooth movement with MOPs [57,59,60,61,62,63,64,69], and two trials tested lasercision [22,66]. In addition, two studies compared corticotomy with corticotomy combined with bone graft procedures [36,42], one compared corticision with corticotomy [45], two compared corticotomy with flapless corticotomy using piezotome (piezocision) [46,47], one tested MOPs and corticotomy [65], two evaluated both MOPs and piezocision [56,58], and one study tested corticotomy, piezocision, and corticotomy versus piezocision [41]. The characteristics of the 39 included trials are presented in Appendix A Table A1, and the extracted data of the included studies are in Supplementary Table S3.

From the six RCT protocols for ongoing studies, one tested lasercision, two investigated the effect of piezocision, one tested MOPs, one evaluated both corticotomy and piezocision, and one studied the effects of both MOP and lasercision. Further information concerning these ongoing research projects is presented in Appendix A Table A2.

### 3.3. Risk of Bias within Studies

Risk of bias assessments for the included studies are summarized in Appendix A Table A3 and Table A4 and Supplementary Tables S4 and S5.

Using the Cochrane risk of bias tool, the risk of bias for the RCTs included in the current systematic review varied from low to high overall. Twelve studies had identified problems with the randomization practices [34,35,36,38,39,40,45,48,49,53,55,65]. Twenty-one of the thirty-three included RCTs with a sufficiently stated randomization process [37,41,42,43,44,46,47,50,51,52,54,56,57,58,59,60,61,62,63,64,66]. In these trials, randomization was performed with coin tosses, computer-generated random numbers, block randomization, or identical, opaque, sealed envelopes to prevent selection bias. The blinding of outcome assessment (detection bias) was possible in 13 trials [29,37,44,45,46,47,48,58,60,61,62,63,66]; participants and operators (performance bias) could be blinded from each surgical technique in four studies [42,43,57,59]. Inadequate reporting was identified and was also associated with deviations from intended interventions and measurement of the outcomes.

Using the ROBINS-I tool, the overall risk of bias for the six CCTs [22,67,68,69,70,71] was judged as being at serious risk. The most severely impacted domains were undetected confounding and classification of interventions, while the risk of systematic discrepancies in the measurement of outcomes also could not be overlooked.

### 3.4. Synthesis of Results

From the total of thirty-three included studies, five articles tested corticotomy [34,35,37,67,71], two examined PAOO [38,39], two studied corticision [43,44], eleven investigated piezocision [40,48,49,50,51,52,53,54,55,68,70], eight evaluated the accelerated tooth movement with the MOPs technique [57,59,60,61,62,63,64,69], and two trials tested lasercision [22,66]. Two studies compared corticotomy with corticotomy combined with bone graft procedures [36,42], one compared corticision with corticotomy [45], two compared corticotomy with flapless corticotomy using piezotome (piezocision) [46,47], one evaluated MOPs and corticotomy [65], two tested both MOPs and piezocision [56,58], and one study evaluated corticotomy, piezocision, and corticotomy versus piezocision [41].

Quantitative data synthesis (meta-analysis) was not conducted due to the heterogeneity of the studies. Therefore, qualitative data analysis was conducted in this review.

#### 3.4.1. Description of Interventions

A corticotomy procedure includes full-thickness flaps elevation, the selective decortication of the buccal and lingual interdental bone to be moved, closure, and suturing the flaps. Only the cortical bone is penetrated or mechanically altered in a controlled surgical approach, and at the same time, the bone marrow is perforated minimally. A modification of this technique is periodontally accelerated osteogenic orthodontics (PAOO) utilizing bone grafts.

Minimally invasive alternatives that create interradicular cuts below each interdental papilla without flap elevation are the following: (a) corticision, where a surgical scalpel and mallet are used to create the cortical bone incision, (b) piezocision utilizing a piezosurgery knife, and (c) lasercision carried out with an Er,Cr:YSGG laser for soft tissue vertical incision in the buccal surface, and the hard tissue laser Er:YAG. for each alveolar perforation.

An additional minimally invasive technique included in the present review is micro-osteoperforations (MOPs), where surgical holes are drilled into the cortical bone using a Propel device, a surgical drill, or mini-implants.

#### 3.4.2. Results of Individual Studies

##### Periodontal Evaluation

The periodontal parameters that were examined throughout the included studies were indices (probing depth, gingival index, plaque index, papillary bleeding index, periodontal index), attachment loss, gingival recession, gingival bleeding, the width of attached gingiva, bone density, alveolar bone height/level, bone width, dehiscence, fenestration, furcation defect, mobility scores, and gingival scar formation. Most of the studies [22,34,35,38,40,41,43,48,49,50,53,54,57,58,59,61,62,66,67,71] that assessed the adverse effects on the periodontium concluded that there were statistically insignificant differences between the experimental and control groups or even within the groups pre- and post-operatively. No significant differences in the probing depth were found between the study groups in a trial by Bahammam et al.; nonetheless, there was a slight improvement in the probing depth values [42]. Sirri et al. reported an increase in the probing depth, the gingival index, the plaque index, and the width of the attached gingiva. However, these changes were not statistically significant between the corticision and control groups [43].

A clinical trial by Khlef et al. compared corticotomy and flapless corticotomy using piezotome (piezocision) and found significant differences between both groups in gingival, papilla bleeding, and plaque indices, before and after orthodontic treatment [48]. Singh and Jayan demonstrated a statistically significant difference in the probing depth, plaque, and gingival indices between the experimental and control groups, with better results shown in the first group following PAOO [39].

Both studies by Charavet et al. observed scar formations in over 50% of the patients in the piezocision group. Significant increases in dehiscence or fenestration in the piezocision and control groups were not recorded [49,50]. On the other hand, according to Agrawal et al., root dehiscence was present at 40% and 30% of the sample after corticotomy and MOP procedures, respectively [65].

Interestingly, Shoreibah et al., while performing PAOO, observed a net increase of approximately 25% in bone density in the group where bone grafting was applied [36]. Bahammam et al. reported a greater increase in bone density in both experimental groups utilizing different bone grafts in comparison with the control group [42]. Singh and Jayan observed that after bone graft placement, the PAOO group presented better results in the probing depth and the gingival and plaque index [39]. However, one trial suggested a significant increase in bone thickness after performing MOPs and corticotomy even without the use of bone grafting [65].

As far as the alveolar bone level is concerned, Raj et al. compared piezocision and conventional orthodontics and reported a greater increase in buccal and mesial bone on the experimental side [51]. However, a statistically significant decrease in the alveolar bone level on the distal surface of the MOPs group was found by Thomas et al. while comparing MOPs and conventional orthodontics. In the same study, a statistically significant increase in probing depth was noted by the end of the trial in both groups [64].

##### Root Resorption

The majority of the studies that assessed the adverse effects on root length and possible root resorption suggested that there was no evidence of substantial apical root resorption [36,39,40,41,42,44,46,49,50,55,57,58,59,60,61,62,63,64,65,68,71]. One trial assessed the amount of root resorption according to CBCT scans after performing corticotomy [37]. This study reported a statistically significant decrease in canine root resorption on the corticotomy side compared with the control side (*p* < 0.05) [Control: Mean ± SD (mm) = 0.53 ± 0.10, 95% CI (0.47, 0.59), corticotomy: Mean ± SD (mm) = 0.24 ± 0.10, 95% CI (0.12, 0.36)].

The study of Abdelqader evaluated the effect of corticotomy and corticision on the root length and concluded at significant canine root resorption in both groups [44]. However, the difference between both interventions was non-significant.

Two trials compared the root resorption after the first premolar extraction between the conventional treatment and piezocision [51,52]. The study of Raj reported significant canine root resorption on the control and experimental sides after an observation period of 6 months [51]. Hartom et al. showed statistically significant root resorption after the completion of en masse retraction in both groups. Only the right and left central incisors and right canine presented statistically significantly (*p* < 0.05) more root resorption in the control group compared to the Piezo group (0.09 ± 0.39, 1.00 ± 0.53, and 1.03 ± 0.63, respectively) [52].

One split-mouth trial that calculated the root length before and after canine retraction using CBCT scans did not report any differences between MOPs or conventional orthodontic treatment (*p* > 0.05) [56]. In the same study, a second comparison was conducted between piezocision and conventional orthodontic treatment. Overall, this study drew the conclusion that piezocision resulted in a significant decrease in canine root length in comparison with both the MOP and control sides after canine retraction (*p* < 0.05). Among the six non-randomized trials, only two of them evaluated root resorption following surgically accelerated orthodontic techniques [69,70]. Chan et al. concluded greater root resorption of 42% on the MOP side compared with traditional orthodontic treatment (MOP: Mean ± SD = 0.576 ± 0.219 mm^3^, Control: Mean ± SD = 0.406 ± 0.168 mm^3^, *p* < 0.001) [69]. The second study [70] tested piezocision against the control and concluded that the piezocision sides presented significantly greater root resorption (*p* < 0.05). In the included studies, there was no reported evidence of the effects of lasercision on the root length.

##### Tooth Vitality

Three randomized [40,47,54] and three non-randomized trials [22,67,71] analyzed the influence of surgically accelerated orthodontic techniques on tooth vitality. Three of them compared corticotomy with conventional orthodontic treatment [47,67,71], two studies investigated piezocision, and one study performed lasercision. All these studies revealed no loss of tooth vitality in any of the examined groups.

## 4. Discussion

### 4.1. Discussion of the Results

Adverse effects on periodontium, root resorption, and tooth vitality following the implementation of surgically accelerated orthodontic techniques were not reported in most of the papers [22,34,35,36,38,39,40,41,42,43,44,46,47,48,49,50,53,54,55,57,58,59,60,61,62,63,64,65,66,67,71]. Slight differences in periodontal parameters and root resorption were shown in a few papers [36,37,39,42,47,51,64,65].

Only one out of thirty-three RCTs had a high quality of scientific evidence [62], twenty-five had some concerns [34,37,38,41,43,44,45,47,48,49,50,51,52,53,54,55,56,57,58,59,60,61,64,65,66], while seven had a high risk [35,36,39,40,42,46,63]. All included CCTs [22,67,68,69,70,71] were classified with a serious risk of bias. The main risk of bias in publications with some concerns and a high risk of bias were selection bias, deviations from intended interventions, and the measurement of the outcomes.

According to the present results, evidence of significant adverse effects on periodontium, root resorption, and pulp vitality is scarce. A meta-analysis of the combined data was not feasible due to the dissimilarities of the retrieved trials. Further research with a sufficiently low risk of bias is required.

### 4.2. Summary of Evidence

Recent evidence suggests that surgically accelerated orthodontic techniques may provide an effective option for shorter orthodontic treatment duration [7,13,24,28]. The orthodontic community has shown great interest in these techniques, which can be inferred from the increasing number of clinical trials in this domain. In the current systematic review, strict eligibility criteria were established to evaluate the effects of surgically accelerated orthodontic techniques on periodontium, root length, and pulp vitality. We investigated 39 randomized and controlled clinical trials that were eligible, with a total of 816 participants.

Corticotomy [34,35,36,37,41,42,45,46,47,65,67,71], PAOO [38,39,42], corticision [43,44,45,46,47], piezocision [40,41,48,52,53,54,55,56,58,68,70], lasercision [22,66], and MOPs [56,57,58,59,60,61,62,63,64,65,69] were performed on children and adolescents between 12 and 19 years of age. Adults aged from 19 to 40 years old underwent corticotomy [35,36,37,41,42,45,46,47,65,67,71], PAOO [38,39,42], corticision [43,44,45,46,47], piezocision [40,41,49,50,51,52,54,55,56,58,68], lasercision [22,66], and MOPs [56,57,58,59,60,62,63,64].

Most of the studies that assessed periodontal parameters post-surgically did not report deleterious effects [22,34,35,38,40,41,43,44,48,49,50,53,54,57,58,59,61,62,66,67,71]. This is in agreement with the findings of several systematic reviews that concluded that the surgically accelerated procedures do not impose a serious risk of harm on periodontal tissues, tooth vitality, and root resorption [24,25,26,28]. The surgical techniques implemented in these reviews were only corticotomy, piezocision, and MOPs. However, a Cochrane review concluded that the data regarding the impact of corticotomy and corticision on periodontal parameters were not clearly reported [7]. In contrast with the already existing systematic reviews, our review evaluates all types of surgically accelerated techniques and their adverse effects, leading to comprehensive results.

Aboul-Ela et al. observed significantly greater gingival index scores on the corticotomy side in comparison with the non-operated side after 4 months [34]. Similar results are reported in two systematic reviews on corticotomy for accelerated orthodontic treatment. They found not only increased gingival index scores at the surgical side 4 months post-operatively but also various complications, such as pain, swelling, subcutaneous hematomas of the face and neck, and dentinal hypersensitivity [24,26].

Piezocision appears to be a promising treatment alternative for accelerated orthodontic tooth movement with no significant periodontal problems [40,47,48,49,50,53,54,59]. In line with our study, a review by Nimeri et al. also did not report evidence of statistically significant periodontal damage after piezocision, and they concluded that it is the least invasive surgical procedure with excellent clinical and aesthetic outcomes [13].

Three of the studies included in the present review compared changes in bone density following corticotomy with and without the application of bone grafts [35,36,42]. Their findings appear to be consistent with the results of Wilcko et al. showing that corticotomy surgery and alveolar augmentation is a safe method and contributes to enhancing the pre-treatment alveolar bone [15]. Even though some studies assessed bone density before and after corticotomy procedures, the number of participants in each study was limited, and their assessment methods varied significantly since different software was used [35,36,42]. Furthermore, these observations were not confirmed by any form of histological examination, and there was no long-term follow-up.

The greater percentage of root dehiscence described in both groups in the study of Agrawal et al. might be attributed to thin buccal bone, implying that this anatomical variation should be considered prior to surgery [65]. The scar formation after piezocision was described in two studies [49,50]. Additional care, namely, sutures, should be provided to patients with high smile lines when this procedure is implemented. However, piezocision may be contraindicated in these patients due to aesthetic issues [50].

Studies implementing piezocision and MOPs reported a high risk of root resorption [51,52,56,69,70]. Furthermore, mild root resorption was demonstrated by Shoreibah et al. [35] in the corticotomy group compared to the control group between the pre-surgical and the 6-month post-surgical values. Nevertheless, the study employed periapical radiographs to measure root resorption, which leads to questionable conclusions, due to the two-dimensional limitations and low accuracy of this assessment method.

Two trials used microtomography to detect root resorption. They reported significantly greater root resorption in areas that underwent MOP [69] and piezocision [70]. However, serious limitations were identified in these experiments regarding the design, due to allocation bias. Moreover, these results should be verified in patients under comprehensive orthodontic treatment since the follow-up period in these trials was only 4 weeks.

Root resorption is a three-dimensional phenomenon, and its extent can be accurately measured with CT and CBCT imaging, which have shown high sensitivity and excellent specificity. Using CBCT to measure external apical root resorption provides reliable results and eliminates the errors produced when two-dimensional radiographs are used [56]. Three studies [41,49,50] investigated root resorption with CT or CBCT and detected no significant difference between the groups. However, another CBCT study reported significantly greater root resorption in both the corticotomy and corticision groups at 5 months [45].

One study [70] reported that piezocision may lead to iatrogenic root damage during surgery when applied in proximity to neighboring roots. Hence, caution should be taken when piezocision is implemented to accelerate tooth movement.

Tooth vitality was briefly assessed in a few studies [22,40,47,54,67,71] and they did not report any case of loss of tooth vitality. However, comparisons between the studies were not achievable due to the different assessment methods that were implemented.

### 4.3. Limitations

Overall, the majority of the included RCTs studies presented a risk of bias of some concerns with inadequate sample size, missing information about randomization, allocation concealment, and the blinding of the outcome assessors. All the included CCTs were considered as being at serious risk of bias due to confounding, intervention assignment affected by knowledge of the outcome, limited information on the blinding of the outcome assessors, and selective outcome reporting. In addition, a long-term follow-up of the response of periodontal tissue, root resorption, and pulp vitality to these surgically accelerated orthodontic procedures was lacking. More than half of the included studies had a split-mouth design that is likely to give biased results if the effect is carried across to the other side of the arch. Bone grafts may also act as a confounding factor. Language restrictions might be an additional limitation. Hence, the results of this systematic review should be taken into careful consideration.

The heterogeneity of the studies mainly concerning the type of outcome assessed and the applied surgically accelerated intervention did not allow a balanced comparison between the results of the included studies, and, therefore, conducting a meta-analysis was not possible.

## 5. Conclusions

Although no major adverse effects following surgically assisted accelerated orthodontic techniques were reported in the available body of literature, currently, there is no scientific evidence to support the presence or absence of clinically meaningful post-operative periodontal side effects, root resorption, and loss of tooth vitality. The results of the present systematic review should be interpreted with caution due to the inadequate sample of participants, short-term follow-up, and unclear safety.

Before these techniques can be proposed in daily clinical practice, reliable conclusions should be provided from further research on their safety. Thus, there is a need for high-quality studies conducted with additional attention paid to increased sample size, improvements in the randomization of participants, the allocation concealment and blinding of the outcome assessments, the follow-up period, the adopted surgical protocol, the type of surgically accelerated orthodontic techniques, the type of examined adverse effects, and the outcome measurement methods.

## Data Availability

The dataset used or analyzed during the proposed systematic review is included in this published article and its Supplementary Information Files.

## Supplementary Materials

The following supporting information can be downloaded at: https://www.mdpi.com/article/10.3390/children9121835/s1, Table S1: Electronic Search Strategy. Table S2: Excluded studies and the reasons beyond exclusion. Table S3: Extracted data of included studies in the systematic review. Table S4: Detailed assessment of included randomized studies with the RoB 2.0 tool. Table S5: Detailed assessment of included non-randomized studies with the ROBINS-I tool.

## Abbreviations

RCTs: randomized clinical trials, CCTs: controlled clinical trials, OTM: orthodontic tooth movement, CAOT: corticotomy assisted orthodontic treatment, RAP: regional accelerated phenomenon, MOPs: micro-osteoperforations, PRISMA: preferred reporting items for systematic reviews and meta-analyses, PAOO: periodontally accelerated osteogenic orthodontics, SD: standard deviation, CT: computed tomography, CBCT: cone beam CT.

## Appendix A

**Table A1:** Characteristics of included studies in the systematic review.

Authors, Publication Year, Study Setting	Study Design	Treatment Comparison	Participants Sample Size, Gender, Age (Years)	Malocclusion	Outcomes	Follow-Up Period
Abbas et al., 2012 Egypt [40]	RCT, parallel study	Alveolar corticotomy using PES vs. conventional OT	Patients (F/M): 8 (8/0) Control: 4 Exp.: 4 Mean age: 22.30 ± 2.26 (19–25)	Class I with minor to moderate mandibular crowding.	No PD > 3 mm, interdental papillae preservation, no GR. No significant reduction in the radiographic height of the crestal bone. No radiographic evidence of any significant apical RR. No loss of tooth vitality.	Until the completion of the decrowding of mandibular teeth.
Abbas et al., 2016 Egypt [41]	split-mouth RCT, multi-arm	Corticotomy vs. piezocision, corticotomy vs. conventional OT, and piezocision vs. conventional OT	Patients (F/M): 20 (NA) Corticotomy group:10 Piezocision group:10 Mean age: 15–25	Class II Division 1 with mild or no crowding.	No differences in the periodontal parameters in either group at the start of canine retraction and after 3 months (*p* > 0.05). No difference between the 2 exp. sides in canine RR. Greater canine RR in the control than the exp. side in both groups.	3 months
Abdelqader 2019 Egypt [45]	split-mouth RCT	Corticision vs. corticotomy	Patients (F/M): 10 (10/0) Control: 10 Exp.: 10 Dropouts: None Mean age: 17–30	Malocclusions requiring maxillary 1st premolar extractions, with subsequent maxillary canine retraction.	Significant canine RR in both groups; however, the difference between both interventions was non-significant.	5 months
Abed and Al-Bustani 2013 Iraq [67]	split-mouth CCT, prospective study	Corticotomy vs. conventional OT	Patients (F/M): 12 (8/4) Dropouts: None Control: 12 Exp.: 12 Mean age: 21.7 (17–28)	Malocclusions requiring maxillary 1st premolar extractions, with subsequent maxillary canine retraction.	No significant difference between pre- and post-surgery on the exp. side (*p* < 0.05) on gingival sulcus depth and tooth vitality. No sign of gingival inflammation according to the BoP index.	1 month
Aboalnaga et al., 2019 Egypt [60]	split-mouth RCT	MOPs vs. conventional OT	Patients (F/M): 18 (18/0) Dropouts: None Control: 18 Exp.: 18 Mean age: 20.5 ± 3.85 (16–30)	Malocclusions requiring bilateral 1st premolar extractions.	No significant difference in RR between the MOP and control sides (*p* > 0.05). No significant difference in RR before and after canine retraction in both groups (*p* > 0.05).	4 months
Aboul-Ela et al., 2011 Egypt [34]	split-mouth RCT	Corticotomy vs. conventional OT	Patients: 15 Dropouts: 2 (1 lost to follow-up, 1 due to poor oral hygiene) Patients (F/M): 13 (8/5) Control: 13 Exp.: 13 Mean age: 19	Class II Division 1 requiring maxillary 1st premolar extractions, with subsequent maxillary canine retraction.	No statistically significant differences (*p* > 0.05) in PI, PD, AL, and GR between the exp. and control sides pre- and postoperatively. GI scores higher on the exp. compared with the control side (*p <* 0.05).	4 months
Agrawal et al., 2018 India [65]	split-mouth RCT	MOPs vs. corticotomy	Patients (F/M): 10 (8/2) Dropouts: None Corticotomy group: 10 MOP group: 10 Mean age: 21.9 ± 2.13 (18–25)	Class I and Class II requiring 1st premolar extractions.	No significant changes in periodontal parameters and root length pre- and post-operatively in both groups (*p >* 0.05). Significant increase after performing both interventions in bone thickness at a coronal level (*p <* 0.05). DF after corticotomy presented in 40% of the sample, while 30% had DF after MOP.	6 months
Aksakalli et al., 2016 Turkey [48]	split-mouth RCT	Piezocision vs. conventional OT	Patients (F/M): 10 (6/4) Dropouts: None Control:10 Exp.: 10 Mean age: 16.3 ± 2.4	Class II requiring maxillary 1st premolar extractions and bilateral canine distalization.	No significant difference in gingival indices and mobility scores between the control and exp. sides pre- and postdistalization (*p* > 0.05).	Until Class I canine relationships were established.
Alkebsi et al., 2018 Jordan [59]	split-mouth RCT	MOPs vs. conventional OT	Patients (F/M): 35 (25/10) Dropouts: 3 subjects were excluded after MOP intervention due to either irregular attendance (Lost to follow-up) or poor oral hygiene. Patients completed the study (F/M): 32 (24/8) Control: 32 Exp.: 32 Mean age: 19.26 ± 2.48 (16 to 24.6)	Class II Division 1 requiring 1st premolar extractions.	No statistically significant difference in PI, GR, GI, AL, and PD between the MOP and control sides at any time point (*p >* 0.05). No adverse effect on periodontal health. No statistically significant difference between the control and MOP sides at baseline T0 (*p* = 0.59) and after 3 months T3 (*p* = 0.48).	3 months
Alqadasi et al., 2019 China [57]	split-mouth, three-dimensional RCT	MOPs vs. conventional OT	Patients (F/M): 8, (F/M NA) Dropouts: None Control: 8 Exp.: 8 Mean age: 15–40	Class II Division 1	No statistically significant difference between the groups in periodontal index, bone height, RR, and PI.	3 months
Alqadasi et al., 2020 China [58]	split-mouth RCT, parallel-group	MOPs vs. conventional OT, piezocision vs. conventional OT	Patients (F/M): 24 MOPs: 12 Piezo: 12 Dropouts: lost to follow-up 3 (2 in MOPs and 1 in Piezo group) Patients completed the study (F/M): 21 (12/9) MOPs: 10 (6F/4M) Piezo: 11 (6F/5M) Mean age: 20.89 ± 4.46 (15–40)	Class II Division 1	No significant differences in either technique regarding RR (*p* = 0.087), buccal (*p* = 0.286), and palatal bone height (*p* = 0.127). MOPs caused more RR than Piezo (*p* = 0.024).	3 months
Arana et al., 2022 Colombia [68]	CCT, prospective study	Piezocision and/or a 3D collagen matrix vs. conventional OT	Patients (F/M): 32 (8/24) Dropouts: None Control: 8 Exp. Group 1: 7 Exp. Group 2: 9 Exp. Group 3: 8 Mean age: 26.9 ± 5.8 (19–38)	Class I or mild Class II or III, and moderate irregularity according to the Little Irregularity Index.	No significant difference in root length observed among the four groups (*p* > 0.05).	Until the completion of orthodontic treatment.
Aristizabal et al., 2016 Colombia [38]	RCT	PAOO vs. conventional OT	Patients (F/M):10 (0/10) Dropouts: None Control: 5 Exp.: 5 Mean age: 18–40 (Control: 28.5 ± 6.3, Exp.: 29.6 ± 9.8)	Class I and II, mild crowding.	Each type of treatment showed no difference in the initial (T1) and final (T2) periodontal conditions.	Treatment time Exp.: 8.2 ± 3.3 months, Control: 13.4 ± 7.3 months.
Bahammam 2016 Saudi Arabia [42]	RCT, prospective study	Corticotomy vs. corticotomy + bovine xenograft vs. Corticotomy + bioactive glass	Patients (F/M): 33 (20/13) Group 1: 11 (7F/4M) Group 2: 11 (6F/5M) Group 3: 11 (7F/4M) Dropouts: 4 lost to follow-up and due to poor oral hygiene. After 9 months: 27 patients follow-up records available Mean age: 21.2 ± 1.43 (18–27)	Class I, moderate crowding.	No significant differences in PD between the study groups (*p* > 0.05). Good interdental papillae preservation, no loss of tooth vitality, and no evidence of significant apical RR at any time interval. Statistically significant greater increase in BD in groups 2 and 3, where grafts were incorporated at T3.	9 months
Bansal et al., 2019 India [61]	RCT, prospective, two-arm, parallel-group study	MOPs vs. conventional OT	Patients (F/M): 30 (16/14) Dropouts: None Control: 15 Exp.: 15 Mean age: 14–19	Mandibular crowding.	No statistically significant differences in RR and marginal alveolar bone height loss around mandibular incisors between the groups (*p* > 0.05).	15 weeks
Chan et al., 2018 Australia [69]	split-mouth CCT, prospective study	MOPs vs. conventional OT	Patients (F/M): 20 (12/8) Dropouts: None Control: 20 Exp.: 20 Mean age: 15.4 (12–25)	Malocclusions requiring 1st premolar extractions.	MOPs resulted in 0.170 mm^3^ or 42% statistically significant greater RR compared with traditional OT (*p* > 0.001).	28 days
Charavet et al., 2016 Belgium [49]	RCT	Piezocision vs. conventional OT	Patients (F/M): 24 (15/9) Control:12 Exp. (piezocision):12 Dropouts: 2 lost to follow-up (1 in each group) failed to attend the post-treatment CT scan. Patients completed the study: 22 Mean age: 30 ± 8	Mild overcrowdings.	Periodontal parameters (recession depth, PD, PI, papilla bleeding index) and the thickness of the buccal alveolar plate, and the buccolingual dimensions of the alveolar crest remained stable between the baseline and treatment completion time points in both groups. Neither GR nor increases in RR were reported in either group. No significant increases in fenestration or dehiscence were observed in either group (*p* = 0.67). Scars were observed in 50% of the patients in the piezocision group, mainly as points (33%) rather than lines (17%).	N/A
Charavet et al., 2019 Belgium [50]	RCT, parallel group	Piezocision vs. conventional OT	Patients (F/M): 24 (15/9) Control: 12 (8/4) Exp.: 12 (7/5) Except for CBCT Control: 11, Exp.: 11 Dropouts: 2 lost to follow-up (1 in each group) failed to attend the post-treatment CBCT scan. Patients completed the study: 22 Mean age: Control: 27 ± 7, Exp.: 29 ± 8	Mild-to-moderate overcrowding.	All periodontal and radiographic parameters remained unchanged from the start to the end of the treatment in both groups. No increase in fenestration (*p* = 0.86) or DF (*p* = 0.12) was observed between the two groups. Minor scars were detected in 66% of cases, mainly as points (58%) rather than lines (8%).	Until treatment completion
Elkalza et al., 2018 Egypt [56]	split-mouth RCT	MOPs vs. conventional OT, piezocision vs. conventional OT	Patients (F/M): 16 (NA) Dropouts: None MOPs: 8 Piezo: 8 Control: 8 Exp.: 8 Mean age: 16–25	Malocclusions requiring maxillary 1st premolar extractions, with subsequent maxillary canine retraction.	No significant difference in canine root length between the MOP and control sides after canine retraction (*p >* 0.05). Statistically significant decrease in root length on the Piezo side compared to the control side (*p* < 0.05).	N/A
Gulduren et al., 2020 Northern Cyprus [62]	split-mouth RCT, single-center, prospective study	MOPs vs. conventional OT	Patients: 20 Control: 10 Exp.: 10 Dropouts: 2 lost to follow-up (due to missing follow-up data) Patients completed the study (F/M): 18 (7/11) Control: 9 Exp.: 9 Mean age: 16.5–23.8	Class II molar relationship, skeletal Class I or mild Class II relationship.	No differences between the groups in PI, GI, PD, and the gingival bleeding of the maxillary 2nd premolars, 1st molars, and 2nd molars. No AL, GR, furcation defect, or mobility observed in these teeth from the start to the end of the experiment. No indication of RR or alveolar bone resorption.	12 weeks
Hatrom et al., 2021 Saudi Arabia [52]	RCT, prospective parallel study	Piezocision vs. conventional en masse retraction	Patients (F/M): 26 (13/13) Control: 13 (7/6) Exp.: 13 (6/7) Dropouts-lost to follow-up: 3 due to mini-screw failure Patients completed the study (F/M): 23 Control: 11 Exp.: 12 Mean age: Control: 20.38 ± 3.64, Exp.: 19.27 ± 3.38	Class II Division I, with mild or no crowding, requiring bimaxillary 1st premolar extractions and subsequent en masse retraction.	Statistically significant RR at the end of en masse retraction in both groups. Only the right and left central incisors and right canine showed statistically significant more RR in the control group compared to the Piezo group (*p* < 0.05).	Until the completion the of en masse retraction phase (mean = 122.74 ± 3.06 days, approx. 4 months)
Karci and Baka 2021 Turkey [53]	split-mouth RCT	Piezocision vs. conventional OT, PRF injection vs. conventional OT	Patients (F/M): 12 (7/5) Control: 12 Exp.: 12 Dropouts: None Mean age: 16.84 ± 0.33	Class II with dentoalveolar protrusion or moderate crowding.	No significant differences in the periodontal readings between the exp. and control sides.	12 weeks
Khlef et al., 2020 Syria [46]	RCT, single-centered, parallel-group	Traditional corticotomy vs. flapless corticotomy (corticision)	Patients (F/M): 40 (36/4) Dropouts: None TCG: 20 FCG: 20 Mean age: TCG: 22.44 ± 3.55 FCG: 21.90 ± 3.60	Class II Division 1, requiring maxillary 1st premolar extractions.	No significant differences in the amount of EARR in maxillary anterior teeth (*p* = 0.31). The proportion of the detected EARR ranged from 1% to 6% of the root length in both corticotomy groups.	Until canines reached Class I relationship with normal overjet and overbite.
Khlef et al., 2022 Syria [47]	RCT, single-centered, two-arm parallel-group	Traditional corticotomy vs. flapless corticotomy (corticision)	Patients (F/M): 38 (35/3) TCG: 19 FCG: 19 Dropouts: 2, 1 in each group Patients completed the study (F/M): 36 (35/3) TCG: 18 FCG: 18 Mean age: TCG: 22.44 ± 3.55 FCG: 21.90 ± 3.60	Class II Division 1, requiring maxillary 1st premolar extractions followed by en masse retraction.	No significant differences in GI, PI, and papillary bleeding between FGC and TCG at T0 and T1 (*p* > 0.017), but significant differences between both groups at T2 (*p* < 0.017). No GR at examined teeth in both groups at T0, T1, and T2. All teeth preserved their vitality following both corticotomies.	Until canines reached Class I relationship with a correct incisor relationship.
Mahmoudzadeh et al., 2020 Iran [66]	split-mouth RCT, parallel study	Lasercision vs. conventional OT	Patients (F/M): 12 (9/3) Dropouts: None Control: 12 Exp.: 12 Mean age:18.91 ± 3.87 (15–30)	Malocclusions requiring bilateral maxillary 1st premolar extractions.	No significant difference in GI between the laser and control groups at baseline and 1 month after the intervention (*p* = 0.55). No significant difference between the laser and control sides in WAG neither before nor after the retraction.	1 month
Patterson et al., 2017 Australia and Greece [70]	split-mouth CCT	Piezocision vs. conventional OT	Patients (F/M): 14 (8/6) Dropouts: None Control: 14 Exp.: 14 Mean age: 16.2 (13.1–19)	Malocclusions requiring maxillary 1st premolars extractions.	Statistically greater RR (*p* < 0.05) on the Piezo sides than the control sides (*p* = 0.029). Piezocision resulted in an average of 0.133 mm^3^, or 44%, increase in RR.	4 weeks
Raj et al., 2020 India [51]	split-mouth RCT, prospective study	Piezocision vs. conventional OT	Patients (F/M): 26 Did not receive the intervention: 2 (1 in each group) Lost to follow-up: 4 (2 in each group due to lack of attendance) Excluded due to incomplete data: 6 (3 in each group) Patients completed the study (F/M): 20 (14/6) Control: 20 Exp.: 20 Mean age: 23.18 ± 1.41 (20–25)	Class II, requiring 1st premolar extractions, with subsequent maxillary canine retraction.	No statistically significant difference in PI. Increase in PD in both groups between the baseline and 6 months and statistically significant increase in RAL (*p* < 0.05). Statistically significant greater increase in buccal and mesial ABL in the Piezo side (*p* < 0.05). Statistically significant increase in RR after 6 months in both groups (*p* < 0.001).	6 months
Ravi et al., 2022 India [55]	split-mouth RCT	Piezocision vs. conventional OT	Patients (F/M): 15 (NA) Piezo (Group II): 15 Control (Group I): 15 Mean age: 18–26	Malocclusions requiring maxillary 1st premolar extractions, with subsequent maxillary canine retraction.	No statistically significant difference in the RR between the two groups.	90 days
Raza et al., 2021 India [37]	split-mouth RCT, single-centered, parallel-group	Corticotomy vs. conventional OT	Patients (F/M): 10 (4/2) Dropouts: None Control: 10 Exp.: 10 Mean age: 18–25	Malocclusions requiring maxillary 1st premolar extractions, with subsequent maxillary canine retraction.	Statistically significant (*p* < 0.05) decreased RR at the corticotomy sides compared with the control sides.	Until the completion of canine retraction. (Exp.: 5.7 months, Control: 7.1 months)
Salman and Ali 2014 Iraq [22]	split-mouth CCT	Lasercision vs. conventional OT	Patients (F/M): 15 (10/5) Dropouts: None Control: 15 Exp.: 15 Mean age: 21.7 (17–28)	Class I or II, requiring bilateral maxillary 1st premolar extractions, with subsequent maxillary canine retraction.	No significant change in gingival sulcus depth around maxillary canines (<4 mm pre- and post-surgery). No pathologic changes in PDL on periapical radiograph; no change in tooth vitality. No sign of gingival inflammation or scar formation.	6 weeks
Shahrin et al., 2021 Malaysia [63]	RCT, prospective, single-center, two-arm parallel study	MOPs vs. conventional OT	Patients (F/M): 30 (25/5) Dropouts: 2 (1 lost to follow-up in the control group, 1 discontinued MOPs due to pregnancy) Patients completed the study: 28 Control: 14 Exp.: 14 Mean age: 22.66 ± 3.27	Moderate maxillary anterior crowding of 5–8 mm.	No significant difference in root length between the MOP and control groups.	6 months
Shoreibah et al., 2012 a Egypt [35]	RCT, prospective, parallel arms	Corticotomy vs. conventional OT	Patients (F/M): 20 (17/3) Dropouts: None Group I (Corticotomy): 10 Group II (Conventional): 10 Mean age: 18.4–25.6	Class I skeletal pattern with moderate mandibular anterior crowding of 3–5 mm.	Six months post-treatment, both groups showed a decrease in PD, which was non-significant. No statistically significant difference in BD between the two groups from baseline to six months post-treatment.	6 months
Shoreibah et al., 2012 b Egypt [36]	RCT, prospective, parallel arms	Corticotomy vs. corticotomy + bioactive glass	Patients (F/M): 20 (16/4) Group I: 10 Group II: 10 Dropouts: 3 lost to follow-up After 6 months: 17 patients follow-up records available Mean age: 24.5	Class I skeletal pattern with moderate mandibular anterior crowding of 3–5 mm.	Six months post-treatment, both groups demonstrated a decrease in PD, which was non-significant. Statistically significant difference between groups in BD from the baseline to six months post-orthodontic treatment. No statistically significant difference in root length in both groups.	6 months
Singh and Jayan 2019 India [39]	RCT	PAOO vs. conventional OT	Patients (F/M): 30 (NA) Dropouts: None Group I (PAOO): 15 Group II (Conventional): 15 Mean age: 18–40	Bimaxillary dentoalveolar protrusion.	Statistically significant difference in PD, PI, and GI between the groups, with Group I displaying better results than Group II. No statistically significant difference in the gingival bleeding index and RR.	Until the completion of retraction. (Group I: 12.7 months, Group II: 21.2 months)
Sirri et al., 2020 Syria [43]	RCT, two-arm parallel-group	Corticision vs. conventional OT	Patients (F/M): 60 (41/19) Dropouts: None Control: 30 (20/10) Exp.: 30 (21/9) Mean age: 21.40 ± 1.63	Mild and moderate crowding (<6 mm according to Little’s index).	No significant differences in the periodontal parameters (PD, PI, GI, WAG). Increase in the PI on the buccal surface in corticision and control groups. Increase in PD on the buccal surface in the corticision and control groups, which were statistically insignificant. Increase in GI on the buccal surface in the corticision and control groups. WAG on the buccal surface in the corticision and control groups.	Until leveling and alignment (Little’s index < 1 mm).
Sirri et al., 2021 Syria [44]	RCT, two-arm parallel-group	Corticision vs. conventional OT	Patients (F/M): 52 (38/14) Dropouts: None Control: 26 (18/8) Exp.: 26 (20/6) Mean age: 21.38	Mild to moderate crowding of the lower anterior teeth (2–6 mm according to Little’s index).	No statistically significant difference was observed between the two groups concerning the overall mean value of EARR after the alignment (*p* = 0.436). No statistically significant difference between the two groups regarding the distribution of DF (*p* = 0.780).	Until leveling and alignment (Little’s index < 1 mm).
Sultana et al., 2022 Malaysia [54]	RCT, single-centered, two-arm parallel-group	Piezocision vs. conventional OT	Patients (F/M): 16 (NA) Piezo: 8 Control: 8 Dropouts: 3 (2 in Piezo, 1 in the control group) due to COVID-19 pandemic restrictions. Patients completed the study (F/M): 13 (NA) Piezo: 6 Control: 7 Mean age: Piezo: 20.83 ± 2.32, Control: 21.14 ± 2.97	Severe anterior maxillary crowding, requiring bilateral 1st premolar extractions (7–9 mm according to Little’s index).	No significant change in PD and AL between T3–T0 in both groups (*p* > 0.05), and all teeth preserved their vitality during the study.	Until complete leveling and alignment
Suryavanshi et al., 2015 India [71]	split-mouth CCT	Corticotomy vs. conventional OT	Patients (F/M): 10 Control: 10 Exp.: 10 Mean age: 18–35	Class II Division 1, large overjet, requiring maxillary 1st premolar extractions	No clinical evidence of GR or any periodontal damage, tooth mobility, or radiographic evidence of RR. No loss of vitality was noted.	Until the completion of canine retraction. 6 months follow-up.
Thomas et al., 2021 India [64]	split-mouth RCT	MOPs vs. conventional OT	Patients (F/M): 33 (24/9) Dropouts: 3 (lost to follow-up) Patients completed the study: 30 Control: 30 Exp.: 30 Mean age: 22.1 ± 2.19 (19–25)	Class I or Class II Division I bilateral maxillary protrusion, requiring 1st premolar extractions, with subsequent maxillary canine retraction.	Statistically significant increase in the PD following 90 days of retraction on both sides. No significant change in AL in intragroup and intergroup comparisons. No significant change in root length between the sides. ABL showed no statistically significant difference in any surfaces.	90 days

**Abbreviations:** RCT: randomized clinical trial, CCT: controlled clinical trial, OT: orthodontic treatment, F: female, M: male, NA: not available, Exp.: experimental group, MOPs: micro-osteoperforations, PAOO: periodontally accelerated osteogenic orthodontics, PES: piezoelectric surgery, Er, Cr: YSGG: erbium, chromium-doped yttrium scandium gallium garnet, DFDBA: demineralized freeze dried bone allograft, TAD: temporary anchorage device, Ni-Ti: nickel-titanium, CBCT: cone beam computed tomography, PDL: periodontal ligament, PD: probing depth, PI: plaque index, GI: gingival index, BoP: bleeding on probing, GR: gingival recession, AL: attachment loss, ABL: alveolar bone level, RAL: relative attachment level, WAG: width of the attached gingiva, BD: bone density, DF: dehiscence formation, EARR: external apical root resorption, RR: root resorption.

**Table A2:** Protocols of the ongoing studies registered at the Clinical.Trials.gov and the Cochrane Library.

Study			Methods		Participants	Interventions		Outcomes	Notes
ID	Setting	Trial Name/Title	Study Design	Treatment Comparison	Sample Size, Gender, Age, Malocclusion	Type and Site of Intervention/Technical Aspects of Interventions & Orthodontic Aspects	Duration/Follow-Up Period	Primary and Secondary	
NCT04631419 Register: Clinical.Trials.gov	Orthodontic department, Faculty of dentistry, Suez Canal University, Ismailia, Egypt	Effects of Flapless Laser Corticotomy in Canine Retraction	RCT, parallel group, split-mouth Single-blind (outcomes assessor)	Flapless laser corticotomy vs. conventional OT (canine retraction)	N = 14 Both (F and M) 18 and older Dental malocclusion, bimaxillary protrusion.	Flapless laser corticotomy will be performed by Er, Cr: YSGG laser as a series of circular holes (3 mm deep) will be created along with the planned position. Canine retraction will be started after this procedure, using a 150 g coil spring and mini-screws as anchorage.	3 months	Primary outcomes: Rate of canine retraction. Secondary outcomes: Canine rotation, 1st molar anchorage loss, root resorption, periodontal condition, pulp vitality.	Status: Active, not recruiting Starting date: 1 June 2018 Completion date: June 2021
NCT05265416 Register: Clinical.Trials.gov	University of Damascus, Damascus, Syrian Arab Republic	Pain and Discomfort and Periodontal Status in Two Acceleration Methods of Canine Retraction	RCT, parallel group Single-blind (outcomes assessor)	Piezocision vs. conventional OT (canine retraction)	N = 58 Both (F and M) 17 to 28 years Class II malocclusion.	Three vertical incisions will be created (3 mm depth and 8–10 mm length) after anesthesia. The cuts will be performed mesial and distal the upper canine as well as at an equal distance from the upper canine and 2nd premolar. Closed nickel-titanium coil springs applying 150 g of force per side will be used for the retraction of the upper canine.	3–4 months	Primary outcomes: change in the levels of pain, discomfort, swelling, levels of eating difficulty, change in the levels of satisfaction. Secondary outcomes: Plaque index, gingival index, bleeding index, Probing depth.	Status: Recruiting Starting date: 22 October 2021 Completion date: 30 November 2022
CTRI/2018/05/013550 Register: Cochrane Library	Department of Orthodontics, Nair Hospital Dental College, Mumbai, India	A study to find out effect of small perforations in gums for faster teeth movement using braces	RCT, parallel group Single-blind (outcomes assessor)	MOPs vs. conventional OT	N = 40 Both (F and M) 18 to 30 years Class I malocclusion, bimaxillary protrusion, less than 4 mm crowding in each arch.	Flapless cortical perforations will be created after alignment leveling. Three MOPs (6 mm deep) will be placed with manual instruments into the maxillary and mandibular interradicular spaces of all anterior teeth including the distal of canine teeth. En masse anterior teeth will then be retracted using conventional sliding mechanics.	18 months	Primary outcomes: Rates of maxillary and mandibular anterior teeth en masse retraction in the intervention and control groups, Total duration of en masse retraction. Secondary outcomes: Root resorption of anterior teeth in both groups, Pain assessment after placement of MOPs.	Status: Open to Recruitment Starting date: 1 May 2018 Completion date: NA
CTRI/2021/05/033657 Register: Cochrane Library	Department of Orthodontics, Coorg Institute of Dental Sciences, Karnataka, India	A study used to determine in which among the two methods of fastest orthodontic tooth movement have more amount of root resorption	RCT, parallel group, split-mouth Open label	Group A: Piezocision vs. conventional OT Group B: Platelet rich plasma (PRP) vs. conventional OT	N = 20 Both (F and M) 18 to 45 years Class I malocclusion indicated for bilateral maxillary 1st premolar extraction.	In group A, retraction will be started with piezocision on the experimental and without piezocision on the control side, respectively. In group B, retraction will be carried out with a submucosal injection of PRP on the experimental side and without PRP on the control side.	6 months	Primary outcomes: Rate of root resorption in the piezocision and PRP groups. Secondary outcomes: Compare the rate of root resorption between the piezocision and PRP groups.	Status: Not Yet Recruiting Starting date: 15 June 2021 Completion date: NA
CTRI/2018/10/015894 Register: Cochrane Library	Department of Orthodontics and Dentofacial Orthopaedics, Surendera Dental College and Research Institute, Sriganganagar, Rajasthan, India	Comparison of different methods of canine movement	RCT, parallel group, split-mouth Single-blind (outcomes assessor)	Group 1: Corticotomy vs. conventional OT Group 2: Low level laser irradiation vs. conventional OT Group 3: Piezocision vs. conventional OT	N = 45 Both (F and M) 14 to 25 years Class I malocclusion with large overjet or bimaxillary protrusion, or Class II Division 1 malocclusion, with large overjet, requiring therapeutic bilateral 1st premolar extractions with the subsequent retraction of canine.	Group 1: full-thickness flap that will be raised on the buccal side. Vertical groove with a 701 straight fissure carbide bur, 2 mm below marginal crestal bone. Horizontal groove 2 mm subapically of canine root apex. Small indentations will be created over the socket of the extracted 1st premolar. Group 3: Interproximal gingival incisions will be created on the mesiobuccal and distobuccal line angles of the canine with a No. 15 blade 2 mm below the papillae. Vertical cortical alveolar incisions (3 mm deep) will be created with an Ultrasonic instrument (BS1 insert Piezotome), 2 mm below marginal crestal bone. Piezotome will be used to remove the bundle bone from the mesial wall of the extracted socket of the 1st premolar. Orthodontic treatment will be started, and after initial alignment, the 1st premolars will be extracted, and respective interventions will be performed to accelerate canine retraction. Canine retraction will be performed using 150 g force applied by Ni–Ti closed coil springs.	63 days	Primary outcomes: Amount of tooth movement, rate of tooth movement, or treatment time. Secondary outcomes: amount of root resorption of canine in the 3 interventions.	Status: Not Yet Recruiting Starting date: 10 May 2018 Completion date: NA
CTRI/2022/01/039459 Register: Cochrane Library	Department of Orthodontics and Dentofacial Orthopedics, Saveetha Dental College and Hospital, India	A clinical trial to compare the speed of alignment of lower front teeth using two different methods of accelerating tooth movement	RCT, parallel group Blinding: N/A	MOP vs. conventional OT Lasercision vs. conventional OT	N = 33 Both (F and M) 16 to 35 years Moderate crowding (Littles irregularity index > 4 mm) requiring therapeutic mandibular 1st premolar extractions.	MOP in lower anterior interdental regions, 4 mm cervical from the tip of interdental gingiva, there will be 4 punctures with a diameter of 1.8–2 mm and the punctures 2 mm apart into the depth of the cortical bone. Lasercision in the lower anterior interdental regions, at a distance of 4 mm cervical from the tip of the interdental gingiva, multiple punctures with laser tip, and the punctures 1 mm apart for up to 6 mm length vertically into the depth of cortical bone.	6–7 months	Primary outcomes: Rate of decrowding in lower anterior regions. Secondary outcomes: Bone changes- cortical bone height and thickness, root resorption.	Status: Not Yet Recruiting Starting date: 20 January 2022 Completion date: NA

**Abbreviations:** RCT: Randomized Clinical Trial, OT: Orthodontic Treatment, F: Female, M: Male, Er, Cr: YSGG: erbium, chromium-doped yttrium scandium gallium garnet, NA: not available, MOPs: micro-osteoperforations, Ni-Ti: nickel-titanium.

**Table A3:** Risk of bias of included RCTs with the RoB 2.0 tool.

Study	Randomization	Deviations from Intended Interventions	Missing Outcome Data	Measurement of the Outcome	Selection of the Reported Result	Overall
Abbas et al., 2012 [40]	Some concerns	Some concerns	Low	High	Low	High
Abbas et al., 2016 [41]	Low	Some concerns	Low	Low	Low	Some concerns
Abdelqader 2019 [45]	Some concerns	Some concerns	Low	Low	Low	Some concerns
Aboalnaga et al., 2019 [60]	Low	Some concerns	Low	Low	Low	Some concerns
Aboul-Ela et al., 2011 [34]	Some concerns	Some concerns	Low	Some concerns	Low	Some concerns
Agrawal et al., 2018 [65]	Some concerns	Some concerns	Low	Some concerns	Low	Some concerns
Aksakalli et al., 2016 [48]	Some concerns	Some concerns	Low	Low	Low	Some concerns
Alkebsi et al., 2018 [59]	Low	Some concerns	Low	Low	Low	Some concerns
Alqadasi et al., 2019 [57]	Low	Some concerns	Low	Low	Some concerns	Some concerns
Alqadasi et al., 2020 [58]	Low	Some concerns	Low	Low	Low	Some concerns
Aristizabal et al., 2016 [38]	Some concerns	Some concerns	Low	Some concerns	Low	Some concerns
Bahammam 2016 [42]	Low	Some concerns	High	High	Low	High
Bansal et al., 2019 [61]	Low	Some concerns	Low	Low	Low	Some concerns
Charavet et al., 2016 [49]	Some concerns	Some concerns	Low	Some concerns	Some concerns	Some concerns
Charavet et al., 2019 [50]	Low	Some concerns	Low	Low	Some concerns	Some concerns
Elkalza et al., 2018 [56]	Low	Some concerns	Low	Low	Low	Some concerns
Gulduren et al., 2020 [62]	Low	Low	Low	Low	Low	Low
Hatrom et al., 2021 [52]	Low	Some concerns	Low	Low	Low	Some concerns
Karci and Baka 2021 [53]	Some concerns	Some concerns	Low	Some concerns	Low	Some concerns
Khlef et al., 2020 [46]	Low	Some concerns	Low	High	Low	High
Khlef et al., 2022 [47]	Low	Some concerns	Low	Low	Low	Some concerns
Mahmoudzadeh et al., 2020 [66]	Low	Some concerns	Low	Low	Low	Some concerns
Raj et al., 2020 [51]	Low	Some concerns	Low	Low	Low	Some concerns
Ravi et al., 2022 [55]	Some concerns	Some concerns	Low	Low	Low	Some concerns
Raza et al., 2021 [37]	Low	Some concerns	Low	Low	Low	Some concerns
Shahrin et al., 2021 [63]	Low	Some concerns	Low	High	Low	High
Shoreibah et al., 2012 a [35]	Some concerns	Some concerns	Low	High	Low	High
Shoreibah et al., 2012 b [36]	Some concerns	Some concerns	Low	High	Low	High
Singh and Jayan 2019 [39]	Some concerns	Some concerns	Low	High	Low	High
Sirri et al., 2020 [43]	Low	Some concerns	Low	Low	Low	Some concerns
Sirri et al., 2021 [44]	Low	Some concerns	Low	Low	Low	Some concerns
Sultana et al., 2022 [54]	Low	Some concerns	Low	Low	Low	Some concerns
Thomas et al., 2021 [64]	Low	Some concerns	Low	Low	Low	Some concerns

**Table A4:** Risk of bias of included non-randomized studies according to ROBINS-I tool.

Study	Bias Due to/in…							
	Confounding	Selection of Participants into the Study	Classification of Interventions	Deviations from Intended Interventions	Missing Data	Measurement of Outcomes	Selection of the Reported Result	Overall
Abed and Al Bustani 2013 [67]	Serious	Low	Low	Low	Low	Moderate	No Information	Serious
Arana et al., 2022 [68]	Low	Low	Serious	Low	Low	Moderate	Low	Serious
Chan et al., 2018 [69]	Low	Low	Serious	Low	Low	Low	Low	Serious
Patterson et al., 2017 [70]	Low	Low	Serious	Low	Low	Low	Low	Serious
Salman and Ali 2014 [22]	Serious	Low	Serious	Low	Low	Moderate	No Information	Serious
Suryavanshi et al., 2015 [71]	Serious	Low	Serious	Low	Low	Moderate	No Information	Serious
