# Supplementary material for: Adverse Effects of Surgically Accelerated Orthodontic Techniques: A Systematic Review
Source: Children (Basel). 2022 Nov 27;9(12):1835. doi: 10.3390/children9121835 (PMC9777195; doi:10.3390/children9121835)
Supplement: Supplementary file 1 [file children-09-01835-s001.zip › children-2053322-supplementary.pdf]

Supplementary Materials

Table S1. Electronic Search Strategy.

Database	Search Strategy	Results
**PubMed**	**Publication Date: from 1/1/2006 until 22/06/2022**<br>**Search Builder: All fields**<br>(("corticotomies"[All Fields] OR "corticotomy"[All Fields] OR "corticision"[All Fields] OR "piezocision"[All Fields] OR "micro-osteoperforations"[All Fields] OR "lasercision"[All Fields]) AND ("periodontium"[MeSH Terms] OR "periodontium"[All Fields] OR "periodontal ligament"[MeSH Terms] OR ("periodontal"[All Fields] AND "ligament"[All Fields]) OR "periodontal ligament"[All Fields] OR "periodontiums"[All Fields] OR ("root resorption"[MeSH Terms] OR ("root"[All Fields] AND "resorption"[All Fields]) OR "root resorption"[All Fields]) OR (("teeth s"[All Fields] OR "teeths"[All Fields] OR "tooth"[MeSH Terms] OR "tooth"[All Fields] OR "teeth"[All Fields] OR "tooth s"[All Fields] OR "tooths"[All Fields]) AND ("vitalities"[All Fields] OR "vitality"[All Fields])))) AND (2006/1/1:2021/10/2[pdat])	222
**MEDLINE via EBSCOhost**	**Publication Date: from 1/1/2006 until 22/06/2022**<br>#1 corticotomy OR corticision OR piezocision OR micro-osteoperforations OR lasercision<br>#2 periodontium OR root resorption OR tooth vitality<br>#3 #1 AND #2	97
**Scopus**	**Publication Date: from 1/1/2006 until 22/06/2022**<br>((((TITLE-ABS-KEY (corticotomy) OR TITLE-ABS-KEY (corticision)) OR TITLE-ABS-KEY (piezocision)) OR TITLE-ABS-KEY (micro-osteoperforations)) OR TITLE-ABS-KEY (lasercision)) AND ((TITLE-ABS-KEY (periodontium) OR TITLE-ABS-KEY (root resorption)) OR TITLE-ABS-KEY (tooth vitality))	136
**Web of Science**	**Publication Date: from 1/1/2006 until 22/06/2022**	150

	(ALL=((corticotom*) OR (corticision*) OR (piezocision*) OR (micro-osteoperforation*) OR (lasercision*))) AND ALL=((periodontium) OR (root resorption) OR (t**th vitality))	
**Cochrane Library**	**Publication Date: from 1/1/2006 until 22/06/2022**<br>#1 corticotomy OR corticision OR piezocision OR micro-osteoperforations OR lasercision<br>#2 periodontium OR root resorption OR tooth vitality<br>#3 #1 AND #2<br>with Publication Year from 2006 to 2022, with Cochrane Library publication date Between Jan 2006 and Jun 2022, in Trials (Word variations have been searched).	55
**Google Scholar**	**Publication Date: from 1/1/2006 until 22/06/2022**<br>Corticotomy OR corticision OR piezocision OR micro-osteoperforations OR lasercision AND periodontium OR root resorption OR tooth vitality	100 most relevant
**Science Direct**	**Publication Date: from 1/1/2006 until 22/06/2022**<br>corticotomy OR corticision OR piezocision OR micro-osteoperforations OR lasercision AND periodontium OR root resorption OR tooth vitality	100 most relevant
**ClinicalTrials.gov**<br>https://clinicaltrials.gov/	**Search Date: 22/06/2022**<br>((corticotomy) OR (piezocision) OR (cortision) OR (micro-osteoperforations) OR (lasercision)) AND ((periodontium) OR (root resorption) OR (tooth vitality))	23
**ISRCTN registry**<br>https://www.isrctn.com/	**Search Date: 22/06/2022**<br>((corticotomy) OR (piezocision) OR (cortision) OR (micro-osteoperforations) OR (lasercision)) AND ((periodontium) OR (root resorption) OR (tooth vitality))	2
**OpenGrey**<br>http://www.opengrey.eu/	**Search Date: 22/06/2022**<br>((corticotomy) OR (piezocision) OR (cortision) OR (micro-osteoperforations) OR (lasercision)) AND ((periodontium) OR (root resorption) OR (tooth vitality))	2

Table S2. Excluded studies and the reasons beyond exclusion.

Study	Reason for exclusion
Ahn HW, Seo DH, Kim SH, Park YG, Chung KR, Nelson G. Morphologic evaluation of dentoalveolar structures of mandibular anterior teeth during augmented corticotomy-assisted decompensation. Am J Orthod Dentofacial Orthop. 2016 Oct;150(4):659-669. doi: 10.1016/j.ajodo.2016.03.027. PMID: 27692424.	Retrospective study
Al-Naoum F, Hajeer MY, Al-Jundi A. Does alveolar corticotomy accelerate orthodontic tooth movement when retracting upper canines? A split-mouth design randomized controlled trial. J Oral Maxillofac Surg. 2014 Oct;72(10):1880-9. doi: 10.1016/j.joms.2014.05.003. Epub 2014 May 14. PMID: 25128922.	Different parameters assessed: "The velocity of space closure was evaluated as the primary outcome variable", "The levels of pain and discomfort were evaluated as the secondary outcome variables".
Cassetta M, Giansanti M, Di Mambro A, Calasso S, Barbato E. Minimally invasive corticotomy in orthodontics using a three-dimensional printed CAD/CAM surgical guide. Int J Oral Maxillofac Surg. 2016 Sep;45(9):1059-64. doi: 10.1016/j.ijom.2016.04.017. Epub 2016 May 10. PMID: 27178968.	Prospective cohort study
Gantes B, Rathbun E, Anholm M. Effects on the periodontium following corticotomy-facilitated orthodontics. Case reports. J Periodontol. 1990 Apr;61(4):234-8. doi: 10.1902/jop.1990.61.4.234. PMID: 2324923.	Case series
Gibreal O, Hajeer MY, Brad B. Efficacy of piezocision-based flapless corticotomy in the orthodontic correction of severely crowded lower anterior teeth: a randomized controlled trial. Eur J Orthod. 2019 Mar 29;41(2):188-195. doi: 10.1093/ejo/cjy042. PMID: 29931294.	Different parameters assessed: "The primary outcome measure was the overall alignment time (OAT) required to complete anterior alignment of the mandibular dental arch." "The secondary outcome measure was Little's Irregularity Index (LII) measured on study models."
Ma Z, Xu G, Yang C, Xie Q, Shen Y, Zhang S. Efficacy of the technique of piezoelectric corticotomy for orthodontic traction of impacted mandibular third molars. Br J Oral Maxillofac Surg. 2015 Apr;53(4):326-31. doi: 10.1016/j.bjoms.2015.01.002. Epub 2015 Jan 28. PMID: 25638568.	Different parameters assessed: "We recorded duration of treatment including exposure and orthodontic traction, and time to the final extraction. Postoperative complications including trismus, swelling, and pain were also noted."
Shahrin AA, Ghani SHA, Norman NH. Effectiveness of microosteoperforations in accelerating alignment of maxillary anterior crowding in adults: A randomized controlled clinical trial. Am J Orthod Dentofacial Orthop. 2021 Dec;160(6):784-792. doi: 10.1016/j.ajodo.2021.04.021. Epub 2021 Aug 25. PMID: 34452786.	Different parameters assessed: "The primary outcome measure was the overall time taken to complete the alignment of the anterior maxillary arch.", "The secondary outcome was the alignment improvement percentage of the anterior maxillary teeth, which was observed up to the sixth month".

Vercellotti T, Podesta A. Orthodontic microsurgery: a new surgically guided technique for dental movement. Int J Periodontics Restorative Dent. 2007 Aug;27(4):325-31. PMID: 17726988.	Case series
Wang B, Shen G, Fang B, Yu H, Wu Y. Augmented corticotomy-assisted presurgical orthodontics of class III malocclusions: a cephalometric and cone-beam computed tomography study. J Craniofac Surg. 2013 Nov;24(6):1886-90. doi: 10.1097/SCS.0b013e3182a245b3. PMID: 24220368.	Orthognathic surgery
Wilcko MT, Ferguson DJ, Makki L, Wilcko WM. Keratinized Gingiva Height Increases After Alveolar Corticotomy and Augmentation Bone Grafting. J Periodontol. 2015 Oct;86(10):1107-15. doi: 10.1902/jop.2015.150074. Epub 2015 Jul 3. PMID: 26138808.	Case-control study
Wilcko MT, Wilcko WM, Pulver JJ, Bissada NF, Bouquot JE. Accelerated osteogenic orthodontics technique: a 1-stage surgically facilitated rapid orthodontic technique with alveolar augmentation. J Oral Maxillofac Surg. 2009 Oct;67(10):2149-59. doi: 10.1016/j.joms.2009.04.095. PMID: 19761908.	Case report

Table S3. Extracted data of included studies in the systematic review.

Authors, publication year	Subject group	Type and site of intervention/ technical aspects of interventions	Orthodontic protocol	Outcome assessment method
Abbas et al. 2012	Piezosurgery/ Alveolar Corticotomies using PES (mandibular anterior teeth)	Full thickness mucoperiosteal flap reflected. Selective decortications performed (both facial and lingual sides) vertical corticotomy cuts made between the roots using PS ultrasonic tips short of the alveolar crest (about 3 mm). Cortical perforation made at selective areas. Bone graft (Bio glass granules) placed over the decorticated areas.<br>Antibiotic (Augmentin) every 8 hours for 5 days after surgery, NSAID (Diclofenac potassium) every 8 hours.	Expansion of the arches to relief crowding and achieve proper mandibular anterior position. Orthodontic activation two weeks post-surgery. Orthodontic readjustments intervals: 1- 3 weeks (averaged 2 weeks).	Method not specified for PD, interdental papillae preservation, GR.<br>Periapical radiographs for height of crestal bone and RR.<br>Test with ice for tooth vitality.
Abbas et al. 2016	● Corticotomy (maxillary canine)<br>● Piezocision (maxillary canine)	● Cort: Full thickness mucoperiosteal flap elevated, corticotomy vertical cuts, made along the mesial and distal aspects of the canine root starting 2 to 3 mm below the alveolar crest, and perforations performed facially along length of maxillary canine's root with piezotome; bundle bone from extraction socket next to upper canine remove with the piezotome.<br>● Piezo: No flap reflection. Interproximal microincisions made through the gingiva 2mm below the papillae on the mesiobuccal and distobuccal line angles of the canine with a number 15 blade in a Bard Parker blade handle. With the same piezotome, the vertical cortical alveolar incisions were created interproximally 2mm below the alveolar crest. Bundle bone from extraction socket next to upper canine removed with the piezotome.	Orthodontic activation in both groups immediately following the interventions. Maxillary canine retraction with 150g force applied by Ni-Ti closed-coil springs. Retraction force readjustments every 2 weeks.	Loe method for PI and GI. Method not specified for PD, AL, and GR.<br>CBCT scans taken before and at the end of canine retraction to assess canine RR.
Abdelqader 2019	Corticision (maxillary canine)	Corticision performed with scalpel and surgical mallet on the mesio-labial and disto-labial side of the maxillary canine.	Orthodontic activation immediately following the intervention. Bilateral canine retraction with 150g force applied by Ni-Ti closed coil spring using TADs as anchorage.	CBCT for RR using Malmgren index.
Abed and Al-Bustani 2013	Corticotomy perforations (maxillary canine)	Buccal flap raised. 3–4 Corticotomy perforations performed mesially and distally to the canine, with a 1.5 mm round bur, spaced 2 mm apart, 3mm deep on buccal side only.<br>Antibiotics (Amoxicillin) and analgesics (paracetamol) prescribed.	Orthodontic activation immediately following the intervention. Maxillary canine retraction with 200g force applied by elastomeric chain. Retraction force readjustments every week.	BoP index for gingival inflammation.<br>No method specified for Gingival sulcus depth.<br>Periapical radiograph

				Electric pulp test for tooth vitality.
Aboalnaga et al. 2019	MOPs (maxillary canine)	3 months after premolar extraction 3 MOPs were performed buccally, using a TAD (1.8 diameter, 8mm length), midway in the extraction space distal to the maxillary canine.	Orthodontic activation immediately following the intervention. Maxillary canine retraction with 150g force applied by Ni-Ti closing coil springs using TADs as anchorage. Retraction force readjustments every 2 weeks.	CBCT scans for RR according to Malmgren index.
Aboul-Ela et al. 2011	Corticotomy perforations (maxillary canine)	Buccal full thickness flap elevated, randomly assigned corticotomy perforations (by No 2-round bur, low-speed hand piece) extended from the lateral incisor to the first premolar area. The depth of the holes approximated the width of the buccal cortical bone.	Orthodontic activation immediately following the intervention. Maxillary canine retraction with 150g force applied by Ni-Ti closed coil spring using miniscrews as anchorage. Retraction force readjustments every week.	Silness and Loe method PI, GI, PD, AL, and GR.
Agrawal et al. 2018	● Corticotomy (maxillary canine)<br>● MOPs (maxillary canine, contralateral side)	● Cort: Full thickness mucoperiosteal flap elevated. With a surgical carbide bur no. 2, vertical grooves were placed in the interradicular space on the mesial and distal side of canine, extended from a point 2–3 mm away from the alveolar crest to a point approximately 4 mm beyond the apices of the roots. Small round perforations of about 2mm made in between the corticotomy cuts and DFDBA was placed.<br>● MOPs: Flapless MOPs created by micro-implants (at least 3 to 4 mm deep and 1.5 mm wide) on the mesial and distal side of canine root.	Orthodontic activation 2 weeks after surgery. Appliance activation every 2 weeks for 3 months.	Method not specified for PD, GR.<br>CBCT scans for bone thickness, RR, and DF.
Aksakalli et al. 2016	Piezocision (maxillary canines)	After the alignment and leveling phases piezocision performed. No flap elevation, 2 vertical interproximal incisions performed (10 mm long) using a No. 15 blade mesial and distal of maxillary canines, 5 mm apical to interdental papilla. Piezosurgery knife (BS1) used to create cortical alveolar incision (3 mm deep).	Orthodontic activation immediately following the intervention. Canine distalization with 150g force applied by elastomeric chains. Distalization force readjustments every 2 weeks.	GI by Silness and Loe method and mobility scores by Muhleman's index.
Alkebsi et al. 2018	MOPs (maxillary canines)	3 MOPs (1.5 mm width and 3 to 4 mm depth) performed using mini-screws 1.5 × 6 mm on the buccal bone distal to the canines at least 6 months after the extractions.	Orthodontic activation 6 months after the extractions. Maxillary canine retraction with 150g force applied by Ni-Ti closed coil spring using mini-screws as anchorage. Retraction force readjustments every week.	Loe method for PI, periodontal index, AL, PD, GI.<br>Periapical radiographs for RR.
Alqadasi et al. 2019	MOPs (maxillary canine)	3 flapless MOPs (1.5–2 mm width, 5–7 mm depth) performed with an automated mini-implant instrumentation on the buccal bone distal to the maxillary canine. MOPs were randomly allocated to either the right or the left side, distal to the maxillary canine.	Orthodontic activation after the premolar extractions and complete alignment stage, canine retraction with 150g force applied by Ni-Ti closing coil springs using TADs as anchorage.	Method not specified for PI, periodontal index.<br>CBCT for RR and bone height.

Alqadasi et al. 2020	● MOPs (maxillary canine)<br>● Piezocision (maxillary canine)	● MOPs: 3 flapless perforations (approximately 1.5-2 mm diameter, 5-7 mm depth) in the middle of the extraction space using an automated mini-implant driver.<br>● Piezo: cut (3 mm length, 3-5 mm depth) was made with piezoelectric instruments at the center of the extraction site (between the canine and second premolar), 2-mm below the crest of the alveolar ridge.	Orthodontic activation upon the completion of alignment and levelling stage (6-8 months after extractions), canine retraction with 150*g* force applied by Ni-Ti closing coil springs using TADs as anchorage. Retraction force readjustments every 28 days.	CBCT for RR, buccal and palatal bone height.
Arana et al. 2022	Piezocision (six mandibular anterior teeth)	Exp. Group 1: orthodontic treatment with piezocision, Exp. Group 2: orthodontic treatment with piezocision and a 3D collagen matrix (Mucograft), Exp. Group 3: orthodontic treatment with a 3D collagen matrix (Mucograft).<br>Flapless vertical and interradicular cuts made in the buccal surface of the maxillary and mandibular arches with piezoelectric scalpel (Piezotome). In Exp. Groups 2, 3 grafting was performed in the lower interincisive zone.	Orthodontic activation: NA	CBCT for RR of lower incisors and canines.
Aristizabal et al. 2016	PAOO (lower and upper teeth)	Mucoperiosteal flaps raised from the buccal surfaces of the lower and upper teeth beyond the apical zones, selective decortications were made, and bone allograft was placed.	Orthodontic activation 2 days following the intervention. Canine retraction with 150*g* force. Retraction force readjustments every 2 weeks	Florida Probe System PD and GR.
Bahammam 2016	PAOO (mandibular anterior teeth)	Full-thickness flaps only labially from canine to canine at the mandible. Corticotomy cuts on the labial cortical bone with stainless steel round bur (number 2), 1–2 mm below alveolar crest and 1-2 mm below teeth apex. Bone grafts: group 1 no graft, group 2 Bovine xenograft (BioOss→), group 3 Bioactive glass (GlassBone→).<br>Antibiotic, analgesic, and diuretic agents for 7 days.	Orthodontic activation 2 weeks after the surgical procedure. Routine orthodontic adjustments every 2 weeks.	William's probe 26 for PD measurements.<br>Periapical radiographic measurements for BD and root length.
Bansal et al. 2019	MOPs (mandibular anterior teeth)	6 flapless MOPs performed using a 1.6 mm × 8 mm mini-implant on the labial aspect in the mandibular anterior region. The depth of perforation was decided by adding gingival tissue depth plus buccal cortical bone depth.	Orthodontic activation immediately following the intervention. Visits every 3 weeks.	CBCT for volumetric RR, marginal alveolar bone height.
Chan et al. 2018	MOPs (maxillary premolar)	1 flapless MOP (2 mm diameter, 5 mm depth) performed using Propel on the mesial and distal aspects in the midroot region of the experimental side of the first premolar root.	Orthodontic activation immediately following the intervention. 150*g* buccal tipping force applied to premolars. After 28 days first premolars of both MOP and control side were extracted.	Microcomputed tomography scan for RR.
Charavet et al. 2016	Piezocision (both arches)	Vertical interradicular cuts (5mm long and 3mm deep) were created below each interdental papilla in both arches.<br>Analgesics as necessary, and chlorhexidine rinses 0.2% 7 for days.	Flapless piezocisions were performed 1 week after the placement of the orthodontic appliances. Routine orthodontic adjustments every 2 weeks.	Method not specified for recession depths, PD, PI, papilla bleeding index, gingival scars.

				CT for RR (according to Malmgren classification), thickness of buccal alveolar plate, buccolingual dimensions of alveolar crest, DF, and fenestration.
Charavet et al. 2019	Piezocision (both arches)	Vertical interradicular cuts (5mm long and 3mm deep) were created below each interdental papilla in both arches.<br>Analgesics as necessary, and chlorhexidine rinses 0.2% 7 for days.	Orthodontic appliances placed 2 weeks prior to Piezocision. Routine orthodontic adjustments every 2 weeks.	CBCT for RR (according to the Malmgren classification), DF and fenestrations.<br>Method not specified for GR, PD, PI, papilla bleeding index and gingival scars.
Elkalza et al. 2018	● MOPs (maxillary canine)<br>● Piezocision (maxillary canine)	● <u>MOPs</u>: 3 MOPs (1.5mm wide, 2 to 3mm deep) performed distal to the maxillary canine using Propel device.<br>● <u>Piezo</u>: Cortical bone incisions created using piezo surgical knife (Piezomed, tip B1) 3mm deep approximately.	Orthodontic activation immediately after the interventions. Canine retraction with 150*g* force applied by Ni-Ti closed coil springs using mini-screws as anchorage. Retraction force readjustments: NA.	CBCT for RR.
Gulduren et al. 2020	MOPs (maxillary molar region)	1st MOP performed at the first day of the distalization treatment (T0) and repeated every 3 weeks for three times. 2 MOPs performed at a depth of 5-6mm between the 2nd premolars and 1st molars, 1st molars and 2nd molars and distal to the 2nd molars, using 1.4 mm width drills (total 6 MOPs each visit).	Distalization of the maxillary molars was performed with 500 *g* force by miniscrew-supported distalization appliances. Reactivation of the appliance every 3 weeks.	Method not specified for PI, GI, PD, gingival bleeding, GR, AL, furcation defect, mobility.<br>Periapical radiographs for RR.
Hatrom et al. 2021	Piezocision (maxillary anterior teeth)	After leveling and alignment, premolars were extracted. During extraction, piezocision decortication performed only labially from canine to canine.	Orthodontic activation 1 week following the intervention. En-masse retraction of the anterior teeth with 250g force applied by Ni-Ti closed coil spring using miniscrews as anchorage. Retraction force readjustments: NA.	CBCT for RR. Association between the change in pulp volume and RR.
Karci and Baka 2021	Piezocision (maxillary canine)	First premolar teeth were extracted, leveling and alignment was completed. No flap reflection, 2 vertical interproximal incisions performed 4 mm apical to the interdental papillae, on the mesiobuccal and distobuccal sides of the maxillary canines using a No 15 blade. A piezosurgery knife was used to create 3 mm-deep cortical alveolar incisions.	Orthodontic activation immediately following the intervention. Maxillary canine distalization with 150g force applied by Ni-Ti closed coil spring using miniscrews as anchorage. Distalization force readjustments every 2 weeks.	PD, PI, and GI evaluated in the T0 and T6 dental models' scans.
Khlef et al. 2020	● Piezocision (maxillary canine)	First premolar teeth were extracted, leveling and alignment was completed.	Orthodontic activation immediately following the intervention. Maxillary canine retraction with 150g force applied	Digital panoramic radiographs for EARR of

	• Corticotomy (maxillary canine)	• Flapless (FCG): Vertical soft-tissue incisions made on the buccal and palatal gingiva, 2 incisions (5 mm long) made 4 mm apical to the interdental papilla, between the maxillary canines and 2nd premolars, and 1 incision between the roots of the 6 maxillary anterior teeth by using a No. 15 blade. Next, a piezosurgery knife (BS1) performed the cortical alveolar incisions (8 mm long, 3 mm deep).<br>• Trad (TCG): full thickness mucoperiosteal flap elevated. 2 vertical cortical alveolar incisions in the place of first premolar extraction and 1 vertical incision between the roots of maxillary anterior teeth were made by the piezosurgery knife (BS1). The vertical incisions were joined by a horizontal incision using the piezosurgery knives (BS2L and BS2R). The depth of the corticotomy was 3 mm, with vertical incisions starting 2-3 mm apical to the alveolar crest.<br><br>Antibiotics (Augmentin) prescribed for 7 days; analgesics (acetaminophen) as necessary.	by Ni-Ti closed coil spring using miniscrews as anchorage. Retraction force readjustments every 2 weeks.	the maxillary anterior teeth.
Khlef et al. 2022	• Piezocision (maxillary canine)<br>• Corticotomy (maxillary canine)	First premolar teeth were extracted, leveling and alignment was completed. Mini-screws (1.6 mm in diameter, 8 mm in length) were inserted between the upper second premolar and first molar, bilaterally.<br><br>• Flapless (FCG): Vertical soft-tissue incisions made on the buccal and palatal gingiva, 2 incisions (5 mm long) made 4 mm apical to the interdental papilla, between the maxillary canines and 2nd premolars, and 1 incision between the roots of the 6 maxillary anterior teeth by using a No. 15 blade. Next, a piezosurgery knife (BS1) performed the cortical alveolar incisions (8 mm long, 3 mm deep).<br>• Trad (TCG): full thickness mucoperiosteal flap elevated. 2 vertical cortical alveolar incisions in the place of first premolar extraction and 1 vertical incision between the roots of maxillary anterior teeth were made by the piezosurgery knife. The vertical incisions were joined by a horizontal incision using the piezosurgery knives (BS2L and BS2R). The depth of the corticotomy was 3 mm, with vertical incisions starting 2-3 mm apical to the alveolar crest.<br><br>Antibiotics (Augmentin) prescribed for 7 days; analgesics (acetaminophen) as necessary.	Orthodontic activation 4 days following the intervention. En-masse retraction with 250g force applied by Ni-Ti closed coil spring using miniscrews as anchorage. Retraction force readjustments every 2 weeks.	PI and GI according to Silness and Loe.<br><br>Papillary bleeding index according to Muhlemann.<br><br>GR index according to Miller.<br><br>Cold test was applied to examine the vitality of the upper teeth using ethyl chloride spray (endo ice) at a temperature of −50°.
Mahmoudzadeh et al. 2020	Lasercision (maxillary canine)	A vertical incision (2-3 mm deep) was carried out with the Er, Cr: YSGG laser in the buccal surface parallel to the mesial and distal root surfaces of the canine, 2 months after the extractions.	Orthodontic activation immediately following the intervention. Canine	Method not specified for GI and WAG.

			retraction with 150*g* force applied by Ni-Ti closing coil springs.	
Patterson et al. 2017	Piezocision (maxillary 1st premolars)	Flapless vertical corticotomy cuts (4-5 mm long, 2-3mm deep) into the buccal cortical plate, mesial and distal to the Piezo premolar, performed with a piezocision blade.	Orthodontic activation immediately following the intervention. 150*g* buccal tipping force applied to premolars. No reactivation. After 4 weeks, premolars of both Piezo and control sides were extracted and scanned.	Microcomputed tomography scans for RR.
Raj et al. 2020	Piezocision (maxillary canines)	After leveling and alignment, piezocision performed. No flap elevation, vertical interproximal incisions performed (10 mm long) using a No. 15 blade mesial and distal of maxillary canines, 5 mm apical to interdental papilla. Cortical alveolar incision (3 mm deep) with Piezosurgery knife (BS1).<br>Antibiotic (Amoxicillin) prescribed for 5 days. Analgesic (ibuprofen) prescribed for 3 days and chlorhexidine rinses 0.2% suggested for 7 days.	Orthodontic activation immediately following the intervention. Canine distalization with 150*g* force applied by Ni-Ti closed coil spring. Distalization force readjustments at 1, 3 and 6 months postoperatively.	PI, PD, RAL evaluated with periodontal probe UNC-15.<br>CBCT for RR and ABL.
Ravi et al. 2022	Piezocision (maxillary anterior teeth)	A small incision was made at mesial and distal to canine, and a piezoelectric handpiece.	Orthodontic activation Force of 150 g per side was applied using elastic chain bilaterally immediately after the 1st premolar extractions.	CBCT for RR.
Raza et al. 2021	Corticotomy (maxillary canine)	Full thickness flap elevation, vertical cuts or perforations using a 2-round bur, 2mm below the alveolar crest extended to canine apex. Graft (DFDBA) placement.	Orthodontic activation immediately following the intervention. Maxillary canine retraction with 150g force applied by Ni-Ti closed coil spring.	CBCT for RR.
Salman and Ali 2014	Lasercision (maxillary canines)	Soft tissue incisions made mesial and distal to maxillary canines by soft tissue laser Er, Cr: YSGG on buccal side only. 4 circular holes made mesially and distally, 2-3 mm apart; each alveolar perforation made with hard tissue laser Er: YAG, approximately 1.5 mm in diameter and 3 mm in depth.	Orthodontic activation immediately following the intervention. Canine distalization with 150*g* force applied by elastomeric chains. Distalization force readjustments intervals: NA	Method not specified for gingival sulcus depth, gingival health.<br>Periapical radiographs for change in PDL.<br>Vitality test for pulp vitality.
Shahrin et al. 2021	MOPs (maxillary anterior teeth)	2 MOPs (1.5mm wide, 3mm deep) performed 3mm apart, at maxillary anterior teeth except midline, using Propel device.	Orthodontic alignment immediately following the intervention. Follow- up assessments at 4-week intervals, MOPs were repeated at every visit until completion of the alignment stage	Periapical radiographs for RR scoring system proposed by Levander and Malmgren.
Shoreibah et al. 2012 a	Corticotomy (mandibular anterior teeth)	Full thickness flap reflected labially from canine to canine at the mandible. Vertical cuts on labial cortical bone, 1-2mm below alveolar crest, extended 1-2 mm below the apices of the teeth, using a small round bur.	Orthodontic activation immediately following the intervention. Orthodontic adjustments every 2 weeks.	William's probe for periodontal parameters. Periapical radiographs for root length and BD.

		Antibiotic, antiedematous drug, and analgesic for 7 days. Chlorhexidine rinses 0.12% for 14 days.		
Shoreibah et al. 2012 b	Corticotomy + bioactive glass (mandibular anterior teeth)	In both groups: Full thickness flaps were reflected labially from canine to canine at the mandible. Vertical cuts on labial cortical bone, 1-2mm below alveolar crest, extended 1-2 mm below the apices of the teeth, using a small round bur. In Group II followed graft placement (bioactive glass).<br>Antibiotic, antiedematous drug, and analgesic for 7 days. Chlorhexidine rinses 0.12% for 14 days.	Orthodontic activation immediately following the intervention. Orthodontic adjustments every 2 weeks.	William's probe for periodontal parameters.<br>Periapical radiographs for root length and BD.
Singh and Jayan 2019	PAOO (maxillary and mandibular anterior teeth)	Full thickness flap reflected labially from canine to canine at the mandible. Vertical cuts on labial cortical bone, 1-2mm below alveolar crest, extended 1-2 mm be-low the apices of the teeth, using a small round bur. Horizontal subapical cut on the labial aspect of maxilla and mandible. DFDBA placement.	Orthodontic activation 1 week after the intervention. Retraction using Ni-Ti closed coil springs and TADs. Orthodontic readjustments intervals: NA	William's probe for periodontal PD.<br>Loe's method for PI and GI.<br>Muhlemann's method for gingival bleeding index.<br>Sharpe's index for RR via periapical radiographs.
Sirri et al. 2020	Corticision (mandibular anterior teeth)	No flap elevation. Incisions using a surgical blade No 15. between the mandibular canine and the lateral incisor roots on each side, and between the roots of the central incisors, 4 mm apart from the gingival papilla. With surgical mallet Corticision applied only once at the three given areas.	Orthodontic activation immediately following the intervention. Routine orthodontic adjustments every 2 weeks.	William's probe for PD.<br>Method not specified for PI, GI, and WAG.
Sirri et al. 2021	Corticision (mandibular anterior teeth)	No flap elevation. Corticision (3-4mm deep, 4-5mm long) was applied with a surgical blade No.15 and a hammer between the mandibular canine and the lateral incisor roots on each side, and between the roots of the central incisors. Corticision applied only once at the three given areas.	Orthodontic activation immediately following the intervention. Routine orthodontic adjustments every 2 weeks.	CBCT for EARR and DF (according to Yang's classification).
Sultana et al. 2022	Piezocision (maxillary anterior teeth)	First premolar teeth were extracted, leveling and alignment was completed. No flap reflection, 7 vertical incisions performed 3 mm apical to the interdental papillae, on the gingiva using a No 15C blade. A piezosurgery knife (BS1) was used to create 3 mm-deep cortical alveolar incisions.	Orthodontic activation immediately following the intervention. Routine orthodontic adjustments every 4-6 weeks.	Williams periodontal probe (Hu Friedy, Chicago, III) for GR and PD.<br>Tooth vitality was recorded using an EPT.
Suryavanshi et al. 2015	Corticotomy (maxillary canine)	Full thickness mucoperiosteal flap elevated, corticotomy vertical holes performed on the buccal cortex, distal to canine and mesial to second premolar, with a 0.5 mm diameter surgical bur. Bur holes connected with a chisel.	Orthodontic activation immediately following the intervention.	Methods not specified for periodontal parameters.<br>Periapical radiographs for RR.

				Electronic pulp vitality tester for tooth vitality.
Thomas et al. 2021	MOPs and piezocision (maxillary canine)	3 MOPs (2 mm wide, 4 mm deep) made with a lance drill attached to a physiodispenser handpiece; placed 3 mm apart vertically on the mesial and distal aspect of the canine root, starting at a point 6 mm apical from the alveolar crest.<br>Analgesics prescribed.	Orthodontic activation immediately after the interventions. Canine retraction with 150*g* force applied by Ni-Ti closed coil springs using micro-implant as anchorage. Retraction force readjustments every 30 days.	UNC-15 probe for PD, RAL.<br>CBCT for canine root length, ABL and Inter-radicular bone width between canine and lateral incisor.

Abbreviations: *NSAID*: Non-steroidal anti-inflammatory drugs, *NA*: not available, *Exp.*.: experimental group, *MOPs*: micro-osteoperforations, *PAOO*: Periodontally accelerated osteogenic orthodontics, *PES*: piezoelectric surgery, *Er, Cr: YSGG*: erbium, chromium-doped yttrium scandium gallium garnet laser, *Er: YAG*: erbium-doped yttrium aluminium garnet laser, *DFDBA*: demineralized freeze-dried bone allograft*, TAD:* temporary anchorage device, *Ni-Ti*: nickel-titanium, *CBCT:* cone beam computed tomography, *PDL*: periodontal ligament, *PD*: probing depth, *PI*: plaque index, *GI*: gingival index, *BoP*: bleeding on probing, *GR*: gingival recession, *AL*: attachment loss, *ABL*: alveolar bone level, *RAL*: relative attachment level, *WAG*: width of the attached gingiva, *BD*: bone density, *DF*: dehiscence formation, *EARR*: external apical root resorption, *RR*: root resorption, *EPT*: electric pulp tester.

Table S4. Detailed assessment of included randomized studies with the RoB 2.0 tool.

Domain	Reference	Abbas et al. 2012	Abbas et al. 2016	Abdelqader 2019	Aboalnaga et al. 2019	Aboul-Ela et al. 2011	Agrawal et al. 2018	Aksakalli et al. 2016
1. Randomization process	1.1	NI	Y	Y	Y	N	Y	NI
	1.2	NI	PY	NI	Y	Y	NI	NI
	1.3	PN	PN	PN	N	N	N	N
	Assessor's judgement	Some concerns	Low	Some concerns	Low	Some concerns	Some concerns	Some concerns
2. Deviations from intended interventions	2.1	PY	PY	PY	Y	PY	PY	PY
	2.2	Y	Y	Y	Y	Y	Y	Y
	2.3	N	N	N	N	N	N	N
	2.4	NA	NA	NA	NA	NA	NA	NA
	2.5	NA	NA	NA	NA	NA	NA	NA
	2.6	NI	NI	NI	NI	NI	NI	NI
	2.7	N	N	N	N	N	N	N
	Assessor's judgement	Some concerns	Some concerns	Some concerns	Some concerns	Some concerns	Some concerns	Some concerns
3. Missing outcome data	3.1	Y	PY	Y	Y	Y	Y	PY
	3.2	NA	NA	NA	NA	NA	NA	NA
	3.3	NA	NA	NA	NA	NA	NA	NA
	3.4	NA	NA	NA	NA	NA	NA	NA
	Assessor's judgement	Low	Low	Low	Low	Low	Low	Low
4. Measurement of the outcome	4.1	N	N	N	N	N	PN	N
	4.2	PN	N	N	N	N	PN	N
	4.3	NI	NI	N	N	NI	NI	N
	4.4	NI	PN	NA	NA	PY	PY	NA
	4.5	PY	NA	NA	NA	PN	PN	NA
	Assessor's judgement	High	Low	Low	Low	Some concerns	Some concerns	Low
5. Selection of the reported results	5.1	PY	Y	PY	Y	Y	Y	Y
	5.2	N	N	N	N	N	N	N
	5.3	N	N	PN	N	N	N	N
	Assessor's judgement	Low	Low	Low	Low	Low	Low	Low
Overall	Assessor's judgement	High	Some concerns	Some concerns	Some concerns	Some concerns	Some concerns	Some concerns

Domain	Reference	Alkebsi et al. 2018	Alqadasi et al. 2019	Alqadasi et al. 2020	Aristizabal et al. 2016	Bahammam 2016	Bansal et al. 2016	Charavet et al. 2016
1. Randomization process	1.1	Y	Y	Y	NI	Y	Y	NI
	1.2	Y	Y	Y	NI	Y	Y	NI
	1.3	N	PN	PN	N	N	N	PN
	Assessor's judgement	Low	Low	Low	Some concerns	Low	Low	Some concerns
2. Deviations from intended interventions	2.1	PY	PY	PY	PY	PY	Y	PY
	2.2	Y	Y	Y	Y	Y	Y	Y
	2.3	N	N	N	N	N	N	N
	2.4	NA	NA	NA	NA	NA	NA	NA
	2.5	NA	NA	NA	NA	NA	NA	NA
	2.6	NI	NI	NI	NI	NI	NI	NI
	2.7	N	N	N	N	N	N	N
	Assessor's judgement	Some concerns	Some concerns	Some concerns	Some concerns	Some concerns	Some concerns	Some concerns
3. Missing outcome data	3.1	N	Y	N	Y	N	Y	N
	3.2	PN	NA	PN	NA	N	NA	N
	3.3	N	NA	N	NA	NI	NA	N
	3.4	NA	NA	NA	NA	NI	NA	NA
	Assessor's judgement	Low	Low	Low	Low	High	Low	Low
4. Measurement of the outcome	4.1	N	N	N	N	PY	N	N
	4.2	N	N	N	N	NA	N	PN
	4.3	N	N	N	NI	NA	N	NI
	4.4	NA	NA	NA	PY	NA	NA	PY
	4.5	NA	NA	NA	PN	NA	NA	PN
	Assessor's judgement	Low	Low	Low	Some concerns	High	Low	Some concerns
5. Selection of the reported results	5.1	Y	NI	Y	Y	Y	Y	NI
	5.2	N	NI	N	N	N	N	NI
	5.3	PN	NI	PN	N	N	N	NI
	Assessor's judgement	Low	Some concerns	Low	Low	Low	Low	Some concerns
Overall	Assessor's judgement	Some concerns	Some concerns	Some concerns	Some concerns	High	Some concerns	Some concerns

Domain	Reference	Charavet et al. 2019	Elkalza et al. 2018	Gulduren et al. 2020	Hartom et al. 2021	Karci and Baka 2021	Khlef et al. 2020	Khlef et al. 2022
1. Randomization process	1.1	PY	Y	Y	Y	Y	Y	Y
	1.2	PY	Y	Y	Y	NI	Y	Y
	1.3	PN	N	N	N	N	N	N
	Assessor's judgement	Low	Low	Low	Low	Some concerns	Low	Low
2. Deviations from intended interventions	2.1	PY	PY	Y	PY	NI	Y	Y
	2.2	Y	Y	Y	Y	Y	Y	Y
	2.3	N	N	N	N	N	N	N
	2.4	NA	NA	NA	NA	NA	NA	NA
	2.5	NA	NA	NA	NA	NA	NA	NA
	2.6	NI	NI	PY	NI	NI	NI	NI
	2.7	N	N	NA	N	N	N	N
	Assessor's judgement	Some concerns	Some concerns	Low	Some concerns	Some concerns	Some concerns	Some concerns
3. Missing outcome data	3.1	N	Y	Y	Y	Y	Y	Y
	3.2	N	NA	NA	NA	NA	NA	NA
	3.3	N	NA	NA	NA	NA	NA	NA
	3.4	NA	NA	NA	NA	NA	NA	NA
	Assessor's judgement	Low	Low	Low	Low	Low	Low	Low
4. Measurement of the outcome	4.1	N	N	N	N	N	PY	N
	4.2	PN	N	N	N	N	NA	N
	4.3	NI	NI	N	Y	Y	NA	N
	4.4	PN	PN	NA	PN	PY	NA	NA
	4.5	NA	NA	NA	NA	PN	NA	NA
	Assessor's judgement	Low	Low	Low	Low	Some concerns	High	Low
5. Selection of the reported results	5.1	NI	PY	PY	Y	Y	Y	Y
	5.2	NI	PN	N	N	N	N	N
	5.3	NI	PN	N	N	N	N	N
	Assessor's judgement	Some concerns	Low	Low	Low	Low	Low	Low
Overall	Assessor's judgement	Some concerns	Some concerns	Low	Some concerns	Some concerns	High	Some concerns

Domain	Reference	Mahmoudzadeh et al. 2020	Raj et al. 2020	Ravi et al. 2022	Raza et al. 2021	Shahrin et al. 2021	Shoreibah et al. 2012 a
1. Randomization process	1.1	Y	Y	NI	Y	Y	NI
	1.2	PY	PY	NI	Y	Y	NI
	1.3	N	N	N	NI	N	N
	Assessor’s judgement	Low	Low	Some concerns	Low	Low	Some concerns
2. Deviations from intended interventions	2.1	Y	PY	PY	Y	Y	PY
	2.2	Y	Y	PY	PY	Y	PY
	2.3	N	N	N	N	N	N
	2.4	NA	NA	NA	NA	NA	NA
	2.5	NA	NA	NA	NA	NA	NA
	2.6	NI	NI	NI	NI	NI	NI
	2.7	N	N	N	N	PN	N
	Assessor’s judgement	Some concerns	Some concerns	Some concerns	Some concerns	Some concerns	Some concerns
3. Missing outcome data	3.1	Y	N	Y	Y	N	Y
	3.2	NA	N	NA	NA	N	NA
	3.3	NA	PN	NA	NA	N	NA
	3.4	NA	NA	NA	NA	NA	NA
	Assessor’s judgement	Low	Low	Low	Low	Low	Low
4. Measurement of the outcome	4.1	N	N	N	N	PY	PY
	4.2	N	N	N	PN	NA	NA
	4.3	N	NI	NI	N	NA	NA
	4.4	NA	PN	N	NA	NA	NA
	4.5	NA	NA	NA	NA	NA	NA
	Assessor’s judgement	Low	Low	Low	Low	High	High
5. Selection of the reported results	5.1	PY	PY	Y	PY	Y	Y
	5.2	N	PN	N	N	N	N
	5.3	N	PN	N	N	N	N
	Assessor’s judgement	Low	Low	Low	Low	Low	Low
Overall	Assessor’s judgement	Some concerns	Some concerns	Some concerns	Some concerns	High	High

Domain	Reference	Shoreibah et al. 2012 b	Singh and Jayan 2019	Sirri et al. 2020	Sirri et al. 2021	Sultana et al. 2022	Thomas et al. 2021
1. Randomization process	1.1	NI	NI	Y	Y	Y	Y
	1.2	NI	NI	Y	Y	Y	Y
	1.3	N	N	N	N	N	N
	Assessor's judgement	Some concerns	Some concerns	Low	Low	Low	Low
2. Deviations from intended interventions	2.1	PY	NI	Y	Y	Y	Y
	2.2	PY	NI	N	Y	Y	Y
	2.3	N	N	PN	N	N	N
	2.4	NA	NA	NA	NA	NA	NA
	2.5	NA	NA	NA	NA	NA	NA
	2.6	NI	NI	NI	NI	NI	NI
	2.7	N	N	N	N	N	N
	Assessor's judgement	Some concerns	Some concerns	Some concerns	Some concerns	Some concerns	Some concerns
3. Missing outcome data	3.1	N	Y	Y	Y	Y	N
	3.2	N	NA	NA	NA	NA	N
	3.3	PN	NA	NA	NA	NA	N
	3.4	NA	NA	NA	NA	NA	NA
	Assessor's judgement	Low	Low	Low	Low	Low	Low
4. Measurement of the outcome	4.1	PY	PY	N	N	N	N
	4.2	NA	NA	N	N	N	N
	4.3	NA	NA	N	N	Y	N
	4.4	NA	NA	NA	NA	N	NA
	4.5	NA	NA	NA	NA	NA	NA
	Assessor's judgement	High	High	Low	Low	Low	Low
5. Selection of the reported results	5.1	Y	Y	Y	Y	Y	Y
	5.2	N	N	N	N	N	N
	5.3	N	N	N	N	N	N
	Assessor's judgement	Low	Low	Low	Low	Low	Low
Overall	Assessor's judgement	High	High	Some concerns	Some concerns	Some concerns	Some concerns

N: no, NA: not applicable, NI: no information, PN: probably no, PY: probably yes, Y: yes

Table S5. Detailed assessment of included non-randomized studies with the ROBINS-I tool.

Domain	Reference	Abed and Al Bustani 2013	Arana et al. 2022	Chan et al. 2018	Patterson et al. 2017	Salman and Ali 2014	Suryavanshi et al. 2015
1. Confounding	1.1	Y	N	N	N	Y	Y
	1.2	N	NA	NA	NA	N	N
	1.3	NA	NA	NA	NA	NA	NA
	1.4	N	NA	NA	NA	N	N
	1.5	NA	NA	NA	NA	NA	NA
	1.6	N	NA	NA	NA	N	N
	1.7	NA	NA	NA	NA	NA	NA
	1.8	NA	NA	NA	NA	NA	NA
	Assessor's judgement	Serious	Low	Low	Low	Serious	Serious
2. Selection of participants into the study	2.1	N	N	N	N	N	N
	2.2	NA	NA	NA	NA	NA	NA
	2.3	NA	NA	NA	NA	NA	NA
	2.4	Y	Y	Y	Y	Y	Y
	2.5	NA	NA	NA	NA	NA	NA
	Assessor's judgement	Low	Low	Low	Low	Low	Low
3. Classification of interventions	3.1	Y	Y	Y	Y	Y	Y
	3.2	Y	Y	Y	Y	Y	Y
	3.3	N	Y	Y	Y	Y	Y
	Assessor's judgement	Low	Serious	Serious	Serious	Serious	Serious
4. Deviations from intended interventions	4.1	N	N	N	N	N	N
	4.2	NA	NA	NA	NA	NA	NA
	4.3	NA	NA	NA	NA	NA	NA
	4.4	NA	NA	NA	NA	NA	NA
	4.5	NA	NA	NA	NA	NA	NA
	4.6	NA	NA	NA	NA	NA	NA
	Assessor's judgement	Low	Low	Low	Low	Low	Low
5. Missing data	5.1	Y	Y	Y	Y	Y	Y
	5.2	N	N	N	N	N	N
	5.3	N	N	N	N	N	N
	5.4	NA	NA	NA	NA	NA	NA
	5.5	NA	NA	NA	NA	NA	NA

	Assessor's judgement	Low	Low	Low	Low	Low	Low
6. Measurement of outcomes	6.1	Y	Y	N	N	PY	Y
	6.2	NI	NI	N	N	NI	NI
	6.3	Y	Y	Y	Y	Y	Y
	6.4	PN	PN	PN	PN	PN	PN
	Assessor's judgement	Moderate	Moderate	Low	Low	Moderate	Moderate
7. Selection of the reported result	7.1	NI	N	N	N	NI	NI
	7.1	NI	N	N	N	NI	NI
	7.3	NI	N	N	N	NI	NI
	Assessor's judgement	NI	Low	Low	Low	NI	NI
Overall	Assessor's judgement	Serious	Serious	Serious	Serious	Serious	Serious
	N: no, NA: not applicable, NI: no information, PN: probably no, PY: probably yes, Y: yes						
